# ALDOC- and ENO2- driven glucose metabolism sustains 3D tumor spheroids growth regardless of nutrient environmental conditions: a multi-omics analysis

**DOI:** 10.1186/s13046-023-02641-0

**Published:** 2023-03-22

**Authors:** Claudia De Vitis, Anna Martina Battaglia, Matteo Pallocca, Gianluca Santamaria, Maria Chiara Mimmi, Alessandro Sacco, Francesca De Nicola, Marco Gaspari, Valentina Salvati, Francesca Ascenzi, Sara Bruschini, Antonella Esposito, Giulia Ricci, Eleonora Sperandio, Alice Massacci, Licia Elvira Prestagiacomo, Andrea Vecchione, Alberto Ricci, Salvatore Sciacchitano, Gerardo Salerno, Deborah French, Ilenia Aversa, Cristina Cereda, Maurizio Fanciulli, Ferdinando Chiaradonna, Egle Solito, Giovanni Cuda, Francesco Costanzo, Gennaro Ciliberto, Rita Mancini, Flavia Biamonte

**Affiliations:** 1 Department of Clinical and Molecular Medicine, Sant’Andrea Hospital, ‘’Sapienza’’ University of Rome, Rome, Italy; 2 Department of Experimental and Clinical Medicine, ‘’Magna Graecia’’ University of Catanzaro, Catanzaro, Italy; 3 Biostatistics, Bioinformatics and Clinical Trial Center, IRCCS ‘’Regina Elena’’ National Cancer Institute, Rome, Italy; 4 Genomic and Post-Genomic Unit, IRCCS Mondino Foundation, Pavia, Italy; 5 SAFU Laboratory, IRCCS ‘’Regina Elena’’ National Cancer Institute, Rome, Italy; 6 Preclinical Models and New Therapeutic Agents Unit, IRCCS ‘’Regina Elena’’ National Cancer Institute, Rome, Italy; 7 Department of Experimental Medicine, Università Degli Studi Della Campania ‘’Luigi Vanvitelli’’, Naples, Italy; 8 Respiratory Unit, Sant’Andrea Hospital, Sapienza University of Rome, Rome, Italy; 9 Department of Neurosciences, Mental Health and Sensory Organs (NESMOS), Sapienza University of Rome, Rome, Italy; 10 Department of Biotechnology and Biosciences, University of Milano-Bicocca, Milan, Italy; 11 Barts and The London School of Medicine and Dentistry, William Harvey Research Institute, Queen Mary University of London, London, E1 2AT UK; 12 Magna Graecia University of Catanzaro, Interdepartmental Centre of Services, Catanzaro, Italy; 13 Scientific Director, IRCCS ‘’Regina Elena’’ National Cancer Institute, Rome, Italy

**Keywords:** Metastasis, Lung cancer, Breast cancer, Glucose metabolism, ALDOC, ENO2, Tumor spheroids, Omics

## Abstract

**Background:**

Metastases are the major cause of cancer-related morbidity and mortality. By the time cancer cells detach from their primary site to eventually spread to distant sites, they need to acquire the ability to survive in non-adherent conditions and to proliferate within a new microenvironment in spite of stressing conditions that may severely constrain the metastatic process. In this study, we gained insight into the molecular mechanisms allowing cancer cells to survive and proliferate in an anchorage-independent manner, regardless of both tumor-intrinsic variables and nutrient culture conditions.

**Methods:**

3D spheroids derived from lung adenocarcinoma (LUAD) and breast cancer cells were cultured in either nutrient-rich or -restricted culture conditions. A multi-omics approach, including transcriptomics, proteomics, and metabolomics, was used to explore the molecular changes underlying the transition from 2 to 3D cultures. Small interfering RNA-mediated loss of function assays were used to validate the role of the identified differentially expressed genes and proteins in H460 and HCC827 LUAD as well as in MCF7 and T47D breast cancer cell lines.

**Results:**

We found that the transition from 2 to 3D cultures of H460 and MCF7 cells is associated with significant changes in the expression of genes and proteins involved in metabolic reprogramming. In particular, we observed that 3D tumor spheroid growth implies the overexpression of ALDOC and ENO2 glycolytic enzymes concomitant with the enhanced consumption of glucose and fructose and the enhanced production of lactate. Transfection with siRNA against both ALDOC and ENO2 determined a significant reduction in lactate production, viability and size of 3D tumor spheroids produced by H460, HCC827, MCF7, and T47D cell lines.

**Conclusions:**

Our results show that anchorage-independent survival and growth of cancer cells are supported by changes in genes and proteins that drive glucose metabolism towards an enhanced lactate production. Notably, this finding is valid for all lung and breast cancer cell lines we have analyzed in different nutrient environmental conditions. broader Validation of this mechanism in other cancer cells of different origin will be necessary to broaden the role of ALDOC and ENO2 to other tumor types. Future in vivo studies will be necessary to assess the role of ALDOC and ENO2 in cancer metastasis.

**Supplementary Information:**

The online version contains supplementary material available at 10.1186/s13046-023-02641-0.

## Background

Metastasis is a multi-step process that includes the degradation and the detachment from the extracellular matrix (ECM) of the primary site, the invasion of vascular and lymphatic vessels, and the formation of secondary tumors in remote sites [1–3]. Among these events, the detachment from the ECM and the survival within the circulation in the absence of cell–cell and cell-ECM stimuli are crucial factors determining metastatic outcome [4, 5].

ECM-independent survival is a stressful event during which cells suffer from loss of integrin-mediated growth signals, cytoskeletal reorganization, diminished nutrient uptake, and increased reactive oxygen species (ROS) production [6]. The vast majority of cancer cells fail to adapt to these damaging events and, consequently, undergo various forms of cell death, such as anoikis, autophagy, and cell cycle arrest [5, 7]. However, a small percentage of cancer cells, provided with stem cell properties and invasion capabilities, by virtue of their powerful ability to adapt, to reprogram cellular energetics and signaling pathways, evade cell death and thus drive tumor progression [8]. To make a few examples, metastatic cancer cells abnormally enhance the autocrine signaling of growth factors, namely fibroblast growth factor (FGF) and epidermal growth factor (EGF), to activate the pro-survival PI3K/Akt, Ras/MAPK, NF-*κ*B, and Rho-GTPase signaling pathways [9]. Moreover, metastatic cancer cells leave the primary site in the form of clusters instead of single units, and clusters have been reported to restrain anoikis by re-establishing cell–cell contacts [2]. Once in the bloodstream, tumor cells closely interact with activated platelets, whose release of tumor grow factor- beta (TGF-beta) also protects against the lack of cell-ECM interactions present in circulation, by inducing a mesenchymal-like phenotype [10, 11]. The activation of platelets also implies the release of fibrinogen and tissue factor, which protect circulating tumor cells against immune clearance [12].

During the last decade, growing evidence highlighted that ECM detachment is tightly associated with drastic cancer cell metabolic alterations, to the point that metabolic dependences may provide potential targets to restrain tumor progression. Metabolic reprogramming is widely recognized as a hallmark of cancer. In this regard, it has been demonstrated that cancer cells preferentially utilize the glycolytic pathway to produce large amounts of lactate even in the presence of oxygen, a phenomenon known as the “Warburg effect”. However, depending on the tumor type and the nutrient environmental conditions, cancer cells may rely on mitochondrial oxidative phosphorylation (OxPhos) or glutamine metabolism to sustain the malignant phenotype. On the other hand, both the presence and the abundance of nutrients within the local microenvironment may determine the metabolic phenotype that cancer cells adopt to accomplish each stage of the metastatic process. Indeed, ECM detachment causes defective glucose utilization, reduces pentose phosphate pathway (PPP), diminishes adenosine triphosphate (ATP) production, and increased ROS generation [13–15]. Despite advances in this field during the last decade, nutrient demands and related mechanisms that sustain the survival of cancer cells following ECM detachment have not been sufficiently elucidated. Moreover, whether these requirements are cancer type-dependent or rather more general phenomena are issues still insufficiently understood.

In this study, we used a multi-omics approach to widely explore the molecular mechanisms utilized for anchorage-independent cancer cell growth in response to a diverse availability of growth factors and nutrients. To this, we set up an in vitro experimental system based on the growth of 3D tumor spheroids derived from lung adenocarcinoma (LUAD) and breast cancer cell lines in customized nutrient- and growth factors-rich or -restricted culture media.

## Methods

### Cell lines and culture conditions

The human H460 and HCC827 LUAD and MCF-7 and T47D breast cancer cell lines were purchased from the America Type Culture Collection (ATCC-LGC Promochem, Teddington, UK). For 2D culture conditions, H460, HCC827 and T47D cells were grown in RPMI1640 medium (Sigma-Aldrich, St. Louis, MO, USA) while MCF7 were grown in Dulbecco’s Modified Eagle’s Medium (DMEM) medium (Sigma-Aldrich, St. Louis, MO, USA). Both media were supplemented with 10% fetal bovine serum (FBS) (Invitrogen, San Diego, CA) and 1% (v/v) penicillin and streptomycin (Sigma-Aldrich, St. Louis, MO, USA). All cell lines were maintained at 37 °C in humidified 5% CO_2_ atmosphere. Cells were passed twice per week using trypsin, thus leading gentle cell detachment. Cell lines were tested for mycoplasma contamination and STR profiled for authentication.

3D tumor spheroids were grown in two different culture conditions: i) a customized nutrient-rich spheroid medium (3D_SM), consisting of DMEM/F-12 (Sigma-Aldrich, St. Louis, MO, USA) supplemented with 0.5% Glucose (Sigma-Aldrich, St. Louis, MO, USA), 2.5 mM L-Glutamine (Thermo Fisher Scientific, Waltham, MA, USA) [16], 2% B-27, 5 μg/ml Heparin, 20 μg/ml Insulin (Thermo Fisher Scientific, Waltham, MA, USA), 20 ng/ml EGF (Thermo Fisher Scientific, Waltham, MA, USA), 20 ng/ml Recombinant Human bFGF (Thermo Fisher Scientific, Waltham, MA, USA), 0.1% Bovine Serum Albumin (BSA) (Sigma-Aldrich, St. Louis, MO, USA) and 1% (v/v) of penicillin/streptomycin 100U/ml, as previously described by Lobello et al. [17]; ii) customized nutrient-restricted RPMI or DMEM culture media supplemented with only 2% FBS (3D_FBS^low^). Overall, the final concentrations of D-Glucose and L-Glutamine in each 3D culture condition are reported in Table 1.

**Table 1:** Concentrations of D-Glucose and L-Glutamine of the cell culture media used for 3D conditions

3D culture condition	Cell line	D-Glucose	L-Glutamine
Sphere Medium (SM)	H460, HCC827, MCF7, T47D	45 mM	4.99 mM
RPMI FBS^low^	H460, HCC827, T47D	25.11 mM	3.99 mM
DMEM FBS^low^	MCF7	11.24 mM	2.05 mM

Briefly, 20,000 cells/mL were resuspended in an appropriate amount of each medium and seeded onto ultra-low attachment plates (Corning Costar, MA, USA) to form 3D structures. After 4 days, the collected tumor spheroids were resuspended in appropriate volume of culture medium and counted by using Leica Thunder Dmi8 microscope according to the following formulas:

$$sphere\;concentration=sphere\;count\;\div counting\;volume\;(\mu L)$$

$$total sphere count=sphere concentration\times total volume$$

Their diameters were measured through the internal image measuring feature normalized to 100 3D spheroids using imaging software Zen (Leica). Data are reported as mean ± Standard Deviation (SD).

### RNA-seq

Total RNA was extracted using Qiazol (Qiagen, IT), purified from DNA contamination through a DNase I (Qiagen, IT) digestion step and further enriched by Qiagen RNeasy columns for gene expression profiling (Qiagen, IT) [18]. Quantity and integrity of the extracted RNA were assessed by NanoDrop Spectrophotometer (NanoDrop Technologies, DE) and by Agilent 2100 Bioanalyzer (Agilent Technologies, CA), respectively. RNA libraries for sequencing were generated in triplicate using the same amount of RNA for each sample according to the Illumina TruSeq Stranded Total RNA kit with an initial ribosomal depletion step using Ribo Zero Gold (Illumina, CA). The libraries were quantified by qPCR and sequenced in paired-end mode (2 × 75 bp) with NextSeq 500 (Illumina, CA).

### RNA-seq bioinformatics analysis

For each sample generated by the Illumina platform, a pre-process step for quality control was performed to assess sequence data quality and to discard low-quality reads. Primary analysis was carried out with Nextflow nf-core/rnaseq pipeline [19, 20]. Secondary analysis, including differential expression analysis, functional enrichment and inter-comparison GO data visualization were entirely carried out with an in-house package, auto-GO (1), which makes use of the DeSeq2, Enrichr and tidyverse [21–23]. Differentially expressed genes (DEGs) were considered strongly regulated with the DESeq2 results table filtered via absolute log_2_(Fold Change) > 1 and padj < 0.05. All the functional enrichment was carried out via the enrichR libraries “GO_Cellular_Component_2021”, “GO_Biological_Process_202” and “KEGG_2021_Human”. Significance for functional cluster was set at padj < 0.1.

### Protein digestion

Protein digestion was performed by filter-aided sample preparation (FASP) as previously described [24]. An aliquot of the digest (50 μL) was purified by SCX StageTips [24]. Peptides were eluted from StageTips using 7 μL of 500 mM ammonium acetate, 20% acetonitrile (v/v). The eluate was mixed with 45 μL of 0.5% formic acid to lower the organic content below 3% before nanoLC-MS/MS analysis. For generating the spectral library, 8 μL aliquots were withdrawn from each sample and pooled into a single sample. The mix was then loaded onto two separate SCX StageTip fabricated by stacking two plugs of SCX material (Empore extraction disks, Millipore) for higher capacity. Stepwise elution in 8 fractions was achieved by adding eluents of increasing ionic strength. Eluents contained 20% acetonitrile, 0.5% acetic acid (except fraction 8) and increasing amounts of ammonium acetate: 40, 70, 100, 150, 200, 250, 350, 500 mM. The eluates of both StageTips were combined, evaporated, resuspended in 20 μL of mobile phase A and analysed by nanoLC-MS/MS.

### NanoLC-MS/MS analysis

NanoLC-MS/MS analysis was performed on EASY1000 LC system coupled to Q-Exactive “classic” mass spectrometer (Thermo Fisher Scientific, Waltham, MA, USA). Peptides were separated using an in-house made analytical column packed with 3 μm-C18 silica particles. A 2 μL aliquot was injected for each sample analysed in data-independent mode (DIA). The analytical system and nanoLC-MS/MS conditions were previously described [24]. Gradient elution of peptides was achieved at 300 nL/min using a 120 min gradient (from 4% B to 28% B in 90 min, then from 28% B to 50% B in 30 min). Mobile phase A consisted of 97.9% water, 2% acetonitrile, 0.1% formic acid, whereas mobile phase B consisted of 19.9% water, 80% acetonitrile, and 0.1% formic acid. The nanoLC effluent was directly electrosprayed into the mass spectrometer in positive ion mode (1800 V). The mass spectrometer operated in DIA mode, using 26 sequential acquisition windows covering an m/z range of 350-1200 [24]. For library generation, the 8 fractions obtained by StageTip fractionation were analysed using identical chromatographic conditions and operating the mass spectrometer in data-dependent mode using a TOP12 method: a full MS scan at resolution of 70,000, with AGC value at 1.0 × 10^6^ and m/z range of 350–1800, followed by 12 MS/MS scans acquired at 35,000 resolutions using AGC value of 1.0 × 10^5^. Normalized collision energy was set at 25%, isolation window was 1.6 m/z and maximum injection time was 120ms for MS/MS scans. Finally, dynamic exclusion was set at 20.0 s. Injected amounts were 4 μL for fractions 1-5 and 8 μL for fractions 6-8.

### Proteomics data processing

Library generation was achieved in Spectronaut Pulsar (Biognosys, v.13) using default parameters [25]. MS/MS spectra were searched against the Uniprot human protein database accessed on May 20^th^, 2020 (74,823 sequences). DIA data analysis was performed on the same platform (Spectronaut) using default parameters. The number of peptides used for quantification was between 1 and 10 (unique peptides), data filtering was based on *q*-value and no normalization factor was adopted in Spectronaut. Then, we used the default Spectronaut long format input to remove low-intensity ions and perform median normalization using *iq* package [26]. The generated protein table with log2 ratios without missing values was used for differential protein expression analysis by *limma* [27]. Differential expressed proteins (DEPs) were selected by an absolute log_2_ |FC|> 1 and based on a *p*-value ≤ 0.01. Finally, DEPs were intersected with DEGs resulting from an absolute log_2_ |FC|> 1 and *p-*value < 0.05 filter. Pathway enrichment analysis was performed using *GSEABase* [28] annotations and *clusterProfiler* [29]. A Benjamini–Hochberg FDR cutoff of 0.05 was used for the analysis.

### Hydrophilic metabolites extraction and quantification

Cell metabolic profiling was conducted on H460 and MCF7 cells grown as 2D, as well as 3D_SM and 3D_FBS^low^ tumor spheroids. A total of 9 sample (3 technical replicates × 3 biological replicates) for each of the 6 cultures (2 in 2D and 4 in 3D) was analyzed. For the whole procedure HPLC-grade solvents and ultrapure Milli-Q water were used. Hydrophilic metabolites extraction was accomplished following the protocol of Yuan et al. [30]. Briefly, hydrophilic metabolites were extracted from 1 × 10^6^ cells by: (i) addition of 4 ml of 80% (vol/vol) methanol:water (cooled to − 80 °C) containing 65 ng of Reserpine as internal standard (IS), (ii) transfer of the cell lysate/methanol mixture to conical tubes and (iii) centrifugation at 14,000* g* for 5 min at 4–8 °C to pellet the cell debris. (iv) The pellet was re-extracted with 0.5 ml of 80% (vol/vol) methanol/water and (v) the obtained supernatants were united and dried by SpeedVac without heating. Cells and cell lysates were maintained refrigerated on dry ice during the extraction procedure. Each dried extract was reconstituted in 100 μL of methanol: water (50:50 v/v) mixture before LC–MS/MS analysis. Five microliters of each sample were injected into the mass spectrometer (QTRAP® 3200 System, Sciex), which includes an HPLC module (Exion LC-100 HPLC, Shimadzu) for quantification of metabolites. A Luna HILIC-NH2 column, 2,6 μm, 50 × 2,1 mm (Phenomenex) coupled with a SecurityGuard Cartridge HILIC-NH2, 2,1 mm column (Phenomenex) was used for chromatographic separation. Mobile phase A was composed of 20 mM ammonium acetate, in water: acetonitrile, 95% (vol/vol); the pH was adjusted to 8 with ammonium hydroxide before the addition of CH_3_CN; mobile phase B was 100% acetonitrile. Oven temperature was 25 °C. The chromatographic gradient, from 85 to 2% mobile phase B in 25 min at 0.35 mL/min flow rate, was adapted by Bajad et al. [31]. The eluting metabolites were analyzed with the mass spectrometer operating in positive and negative mode, using the Multiple Reaction Monitoring (MRM) approach for targeted profiling. Optimized electrospray ionization parameters were electrospray voltage of respectively 5000/-4500 V in positive/negative mode, temperature of 350 °C, curtain gas of 30 psi, nebulizer gas (GS1) and auxiliary gas (GS2) of 40 and 40 psi, respectively. Dwell time for each MRM transition was 5 ms. Compound dependent parameters, i.e., Declustering Potential (DP), Entrance Potential (EP), Collision Cell Entrance Potential (CEP), Collision Energy (CE) and Collision Cell Exit Potential (CXP) were adapted from an internal Sciex report [32]. and validated on a subset of 24 commercial molecular standards, including all classes of target metabolites. The instrument was mass calibrated with a mixture of polypropylene glycol (PPG) standards. Quality controls and carry-over checks were included with each samples batch. Acquisition was performed by Analyst 1.6.3 software (Sciex).

### Metabolomic data processing

All statistical and correlation analyses were done using MetaboAnalyst 5.0 [33]. Data were normalized versus the IS Reserpine. Hierarchical cluster analysis was performed after autoscaling of data, selecting Euclidean Distance as similarity measure parameter and Ward’s linkage as clustering algorithm. Student’s t test or one-way ANOVA test, followed by post-hoc analyses, were used to compare the relative concentration of metabolites respectively among two or more groups; the *p*-value significance threshold was set at 0.05.

### 3D cell viability assay

Cell titer-Glo 3D (Promega) was used to determine viability of 3D cells plated in single wells of a 96 well ultra-low-attachment culture plate. The assay was performed in triplicate according to manufacturer’s instructions. Samples were read on the GloMax Explorer Luminometer (Promega) [34].

### Scanning Electron Microscopy (SEM) analysis

Glutaraldehyde-fixed samples were rinsed with a cacodylate buffer and then dehydrated with an increasing ethanol percentage (30–90% in water for 5 min, twice 100% for 15 min), treated in a Critical Point Dryer (EMITECH K850), sputter coated with platinum-palladium (Denton Vacuum DESKV), and observed with Supra 40 FESEM (Zeiss).

### ALDOC and ENO2 transient silencing

siRNA transfections were performed as previously described [35, 36]. For each gene we used three different siRNAs: siENO2 (Assay ID s4685, Assy ID 10894, Assy ID 121347); ALDOC (sc-270351, Santa Cruz) and (Assay ID 15795 and Assay ID 121526, Thermo Fisher Scientific, Waltham, MA, USA ). Control siRNAs were purchased from (Thermo Fisher Scientific, Waltham, MA, USA). DNA transfections were performed with Lipofectamine 2000 (Thermo Fisher Scientific, Waltham, MA, USA) according to the manufacturer's instructions.

### RNA extraction, cDNA synthesis and real time PCR

RNA was harvested with TRIzol (Thermo Fisher Scientific, Waltham, MA, USA) as previously described [37–43]. Total RNA (1 μg) was digested with gDNAse Eraser and reverse-transcribed with PrimeScript RT reagent Kit, (Takara Bio Inc). The expression levels of *ALDOC* and *ENO2* were analyzed by using 7500 Step One Plus (Applied Biosystems). The primer sequences used are: *ALDOC* forward 5-CATTCTGGCTGCGGATGAGTC-3, reverse 5-CACACGGTCATCAGCACTGAAC-3; *ENO2* forward 5-AGCCTCTACGGGCATCTATGA-3, reverse: 5-TCAGTCCCATCCAACTCC-3. H3 was included as housekeeping for normalization of real time data [44].

### Intracellular glucose and lactate quantification assays

Analysis of intracellular glucose and lactate quantities were performed by using Glucose-Glo assay and Lactate-Glo Assay, respectively (Promega, Madison, WI, USA). Briefly H460 and MCF7 cells were seeded in 6-well plates and then transfected with the two siRNAs specific for ALDOC and ENOs. After transfection, both cell lines were cultured in 96-well ultra-low-attachment plates. Analysis was performed through GloMax Explorer Luminometer (Promega). Data were normalized to cell number. Analyses were performed in triplicate and results are reported as mean ± SD.

### Extracellular lactate quantification assays

The quantification of L-lactic acid present in the culture media was performed by using Emogas analyzer Gem 5000, according to the manufacturer’s instructions. The amount of L-lactic acid produced by the cells in each sample was calculated subtracting the amount of L-lactic acid in the media (without cells) from the amount of lactate in the media from each sample.

### Statistical analysis

Data were analysed in GraphPad Prism 9. Comparison of more than two groups was performed using one-way ANOVA analysis. Student *t* test was used for two-groups comparisons. A *p*-value < 0.05 was considered statistically significant. Integrated analysis of RNA-seq and Proteomics was carried out via the novel STAR protocol presented by Yang et. al [45] which employs a mixture of differential analysis techniques and custom R statistical mining on both datasets.

## Results

### Design and setting of the study

**Fig. 1:**
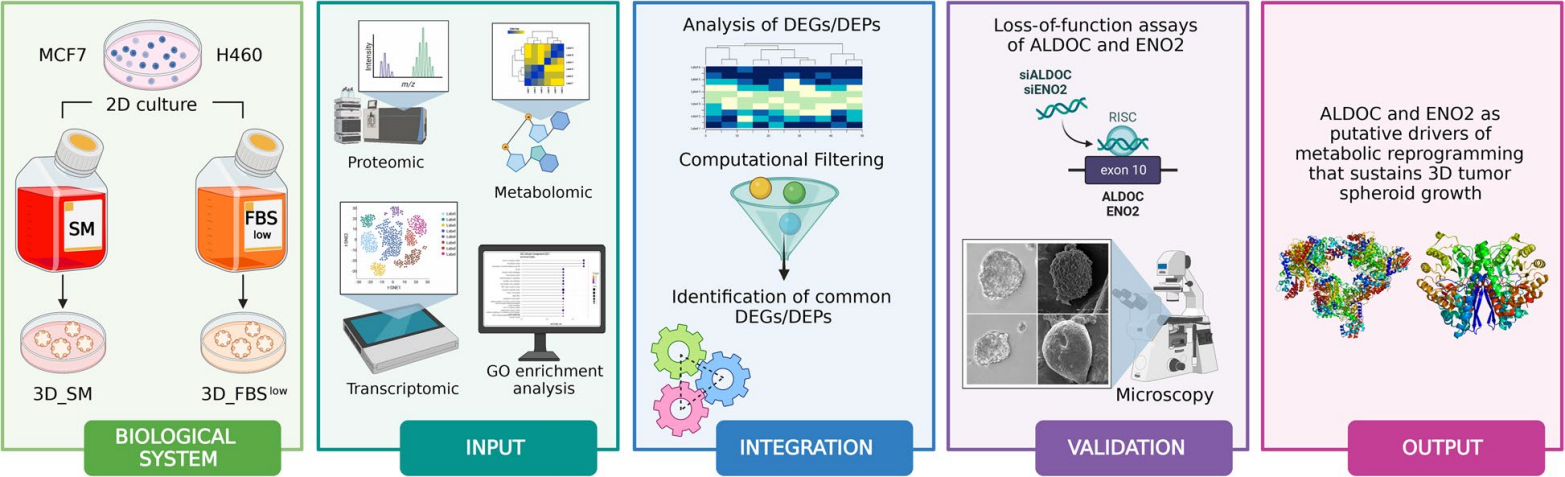
Workflow of the multi-omics integrative analysis. Biological System: H460 LUAD and MCF7 breast cancer cell lines were cultured in 2D and 3D conditions. 3D tumor spheroids were grown either in a nutrient-rich (sphere medium, SM) or in a nutrient-restricted (FBS^low^) culture media. Input: a total of 6 samples (H460 2D, H460 3D_SM, H460 3D_ FBS^low^, MCF7 2D, MCF7 3D_SM, MCF7 3D_ FBS^low^) were characterized through transcriptomic, proteomic and metabolomic analyses; differentially expressed genes (DEGs) and differentially expressed proteins (DEPs) between 3D *vs* 2D samples were further analyzed by using Gene Ontology (GO). Integration: DEGs and DEPs were integrated to identify a common signature of DEGs and DEPs in all the 2D to 3D transitions. ALDOC and ENO2 were found up-regulated in all 3D *vs* 2D culture conditions. Validation: siRNA-mediated knock down of ALDOC and ENO2 were performed to functionally validate the effects of these two enzymes on 3D tumor spheroids growth. Output: ALDOC and ENO2 represent putative drivers of the metabolic reprogramming responsible for the sphere-forming ability of H460 and MCF7 cells

This study aimed to gain insight into the molecular mechanisms allowing cancer cells to survive and proliferate under detached conditions, regardless of both tumor-intrinsic variables and nutrient culture conditions. To this purpose, we used 3D tumor spheroids as i*n vitro* experimental models to mimic anchorage-independent cancer cell growth as well as to mimic fluctuation in nutrient and oxygen availability that cells undergo as tumor mass grows and expands in vivo*.* 3D tumor spheroids derived from LUAD, and breast cancer cell lines were grown in two culture conditions: i) sphere medium (SM), which mimics an environment rich in major nutrients (glucose and L-glutamine) and growth factors (Epidermal Growth Factor, EGF, and basic Fibroblast Growth Factor, bFGF) and ii) RPMI or DMEM FBS^low^, supplemented with only 2%FBS, mimicking a nutrient-restricted culture condition. A wide multi-omic approach, based on the integration of transcriptomic, proteomic, and metabolomic analyses, was used to identify the common molecular changes occurring during all the transitions from adherent 2D to 3D cultures, regardless of the tumor type and nutrient culture availability. Small interfering RNA-mediated loss of function assays were used to validate the role of the identified differentially expressed genes and proteins in LUAD and breast cancer cell lines (Fig. 1).

### RNA-seq analysis highlights that H460 and MCF7 require the rewiring of genes involved in the metabolic programmes to grow in 3D culture conditions

**Fig. 2:**
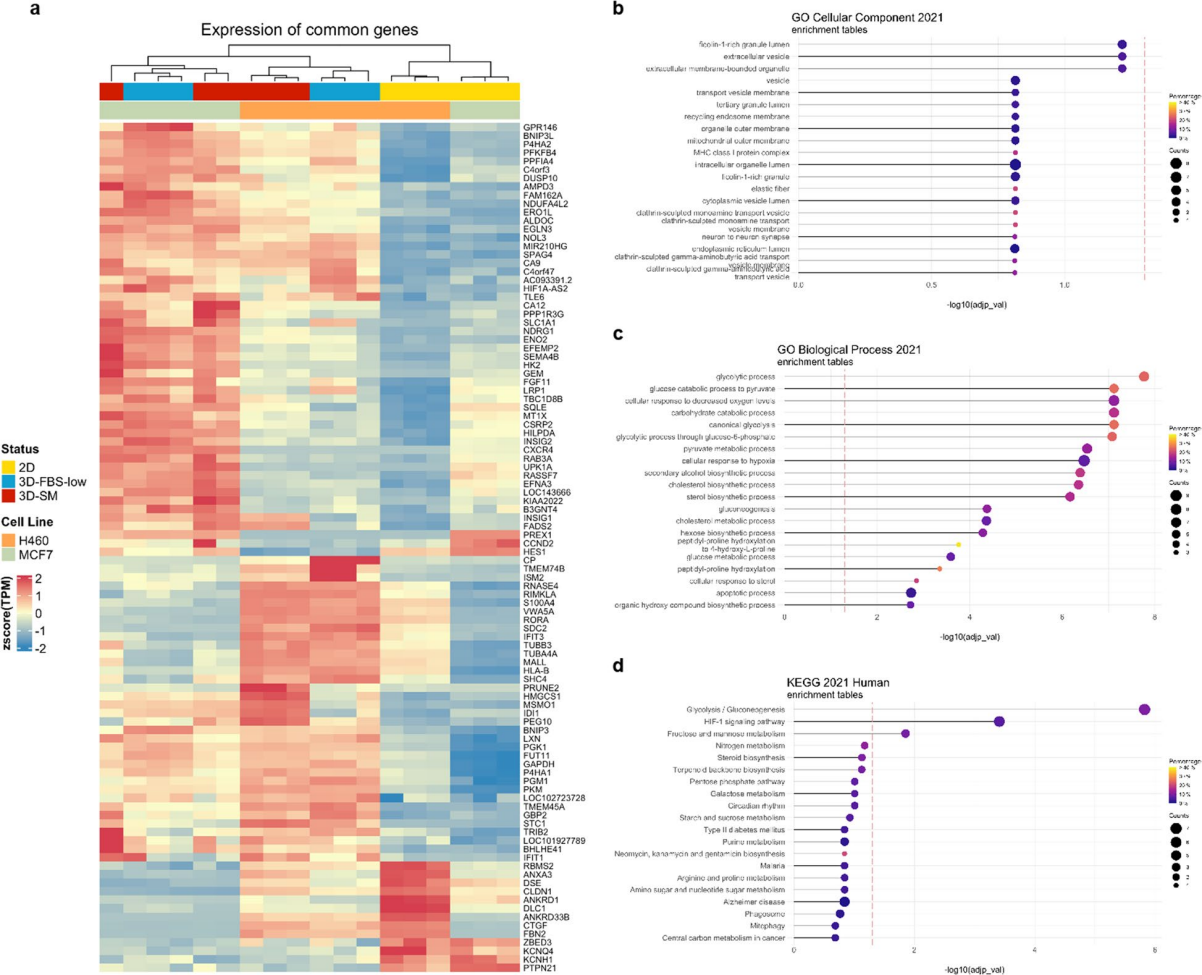
Transcriptomic analysis of H460 and MCF7 cell lines grown in 2D and 3D culture conditions. **A** Heatmap of 100 DEGs in all 3D vs 2D conditions of both cell lines. Color intensity is proportional to the magnitude of changes. Relative expression levels are shown in red (upregulation) and blue (downregulation).** B** GO analysis of cellular component, **C** biological process, and **D** KEGG pathway analysis of DEGs in all 3D *vs* 2D conditions of both cell lines. The dot size denotes the number of DEGs, while colors correspond to the adjusted *p*-value range

Cancer cells grown in 3D cultures show distinct gene expression patterns when compared to the same parental cells grown in 2D conditions [46–48]. Indeed, differential extracellular interactions with ECM as well as different nutrient availability within 3D models change intracellular signal transduction, culminating in the activation of a unique set of transcription factors and in significant changes of the transcriptomic profiles [49, 50]. Here, we first shed light on the transcriptional reorganization associated with 3D cell growth in nutrient-rich or nutrient-restricted culture conditions. H460 and MCF7 cells (1.5 × 10^4^/mL) were grown in non-adherent conditions either in nutrient-rich sphere medium (SM) or in a nutrient-restricted culture medium (FBS^low^). After 4 days, which was previously established as the optimal time frame to collect first generation of tumor spheroids [51], we performed RNA-seq analysis of H460 and MCF7 grown either as a monolayer (2D) or as 3D_SM and 3D_ FBS^low^. Overall, differential expression analysis (DEA) highlighted a total of 2169 DEGs when comparing H460 2D *vs* 3D_SM and 1478 DEGs when comparing H460 2D *vs* 3D_FBS^low^ (Table S1, in Additional file 1). For MCF7 cells, 1925 DEGs emerged from the comparison between 2D *vs* 3D_SM while 2222 DEGs resulted from 2D *vs* 3D_FBS^low^ (Table S1, in Additional file 1). Then, we sought to find a common transcriptional signature associated with 3D tumor spheroid growth, namely genes and processes significantly up or down regulated in the transition from 2 to 3D, regardless of the cell type and the culture media utilized. A signature of 100 genes was found to be commonly regulated in all the systems; among these, 84 genes were commonly up regulated while 16 were commonly down regulated in all 3D *vs* 2D conditions (Fig. 2A). Interestingly, functional enrichment analysis on the common DEGs highlighted that among the enriched biological processes, most of them (17 out of 20) were associated with cellular metabolism. In particular, *Glycolysis/Gluconeogenesis* appeared the most consistently enriched metabolic pathway because of the up regulation of 6 out of 10 glycolytic enzymes (hexokinase 2, *HK2;* pyruvate kinase muscle isozyme, *PKM*; phophoglycerate kinase 1, *PGK1; aldolase C, ALDOC; enolase 2, ENO2;* glyceraldehyde 3-phosphate dehydrogenase, *GAPDH)* and of phosphoglucomutase 1 (*PGM1)*. Notably, both *HK2* and *PKM* encode for muscle-specific isoenzymes involved in the regulation of two irreversible steps of glycolysis [52]. *ALDOC* and *ENO2* encode for neuronal-specific aldolase and enolase isoforms [53, 54]. HK2 catalyses the first priming and irreversible reaction of glycolysis, the conversion of the substrate glucose into glucose-6-phosphate, ALDOC is the key enzyme of the fourth step of glycolysis, during which fructose -1,6-bisphosphate is converted to gylceraldehydes-3-phosphate (G3P) and dihydroxyacetone phosphate (DHAP). GAPDH, PGK1, ENO2, and PKM catalyze 4 out of the 5 reactions of the energy-releasing phase of glycolysis [55, 56]. PGM1 belongs to the phosphohexose mutase family and catalyzes the transfer of phosphate between the 1 and 6 positions of glucose; as such, it is involved in both the synthesis and degradation of glycogen [53, 57–59]. As the second most significantly affected biological process in the 2D to 3D transition, the cellular response to hypoxia (*HIF-1 signalling pathway*) was enriched by the up regulation of *BNIP3, BNIP3L, EGLN3, FAM162A, HILPDA, PGK1, RORA, NDRG1* (Fig. 2B-C) (Tables S2-3, in Additional files 2 and 3). In addition to the glycolytic enzyme *PGK1*, *EGLN3* is a member of the 2-oxoglutarate (2OG)–dependent dioxygenases family responsible for the prolyl hydroxylation of HIF-1//2 and for the regulation of cell apoptosis in response to hypoxia [60]. Similarly, *BNIP3, BNIP3L*, and *FAM162A* are involved in the regulation of cell death in response to hypoxic conditions [61, 62]. In particular, the BH3-only proapoptotic genes BNIP3 and BNIP3L enhance autophagy and, in particular, mitophagy to overcome cell death and guarantee survival under hypoxic conditions [63]. N-myc downstream-regulated gene-1 (*NDRG1*) is a hypoxia inducible-protein involved in the p53-mediated activation of the caspase cascade; furthermore, it influences the epithelial to mesenchymal transition (EMT) as it is required for the vesicular recycling of e-cadherin and for the cadherins switching [64, 65]. The hypoxia-inducible and lipid droplet-associated protein *HILPDA* is known to promote lipid droplets formation in response to hypoxia as well as to autophagic flux induced by nutrient deprivation [66]. *RORA* is a hypoxia-induced member of the retinoic acid-receptor-related orphan receptor α superfamily; unlike the other members of this family, RORA binds to the promoter of cell cycle-related genes and N-myc, thus affecting cell growth and tumorigenesis [67]. Finally, the GO cell component analyses highlighted that *PKM, ALDOC, AMPD3, PGM1, EFEMP2,* and *RAB3A*, are up-regulated in all 3D *vs* 2D culture conditions, consistently enriched the *ficolin-1-rich granule lumen* and *extracellular vesicles* (EVs) (Table S4, in Additional file 4). Together with the already described *PKM*, *ALDOC* and *PGM1*, the Adenosine Monophosphate Deaminase 1 (*AMPD3*), encoding for the red blood cells (RBC)-specific member of the adenosine monophosphate (AMP) deaminase family, catalyzes the irreversible hydrolytic deamination of AMP to inosine monophosphate (IMP), thus it is involved in purine nucleotide, uric acid, and carbohydrate metabolism [68]. Recent reports indicate that, in RBCs, AMPD3 can be activated by the increased intracellular levels of ROS and calcium, along with decreased intracellular pH [69]. The exact role of AMPD3 in cancer is instead still unclear; however, since it controls the intracellular levels of AMP, it is reasonable to hypothesize that it might affect AMP-activated protein kinase (AMPK). AMPK is largely recognized as a key energy sensor. In response to diverse stressors, such as glucose starvation, hypoxia, and oxidative damage, it activates ATP-producing pathways [70]. In agreement, according to several studies, AMPK deficiency renders cancer cells more vulnerable to the stresses induced by cell detachment [71]. *EFEMP2* (EGF Containing Fibulin Extracellular Matrix Protein 2) gene encodes for a member of fibulin glycoprotein family, involved in the stabilization of the ECM structure; indeed, it is necessary for elastic fiber formation, and it is involved in collagen fibril assembly. So far, the role of EFEMP2 in tumorigenesis is found to be “context-specific”; indeed, while in cervical cancer, ovarian cancer, and glioblastoma it has been associated with tumor progression and poor prognosis, in endometrial cancer it has been found to inhibit EMT, tumor invasion and metastasis [72]. Finally, *Rab3A* belongs to the small Ras-like GTPase superfamily and functions as a key regulator in transporting cellular products into secretory vesicles and lysosomes [73]. Normally Rab3A is predominantly expressed in the neural system; however, it has been found aberrantly overexpressed in breast cancer where it is associated with a more malignant phenotype and in hepatocellular carcinoma where, instead, it inhibits metastasis via enhancing mitochondrial oxidative metabolism [74].

Overall, RNAseq data suggest that the ability of cancer cells to survive and grow in 3D culture conditions requires the rewiring of intracellular metabolic pathways and the control of redox homeostasis most likely in response to the decreased oxygen levels.

### Proteomic analysis confirms that H460 and MCF7 cells reprogram their glucose metabolism to survive in all 3D culture conditions

**Fig. 3:**
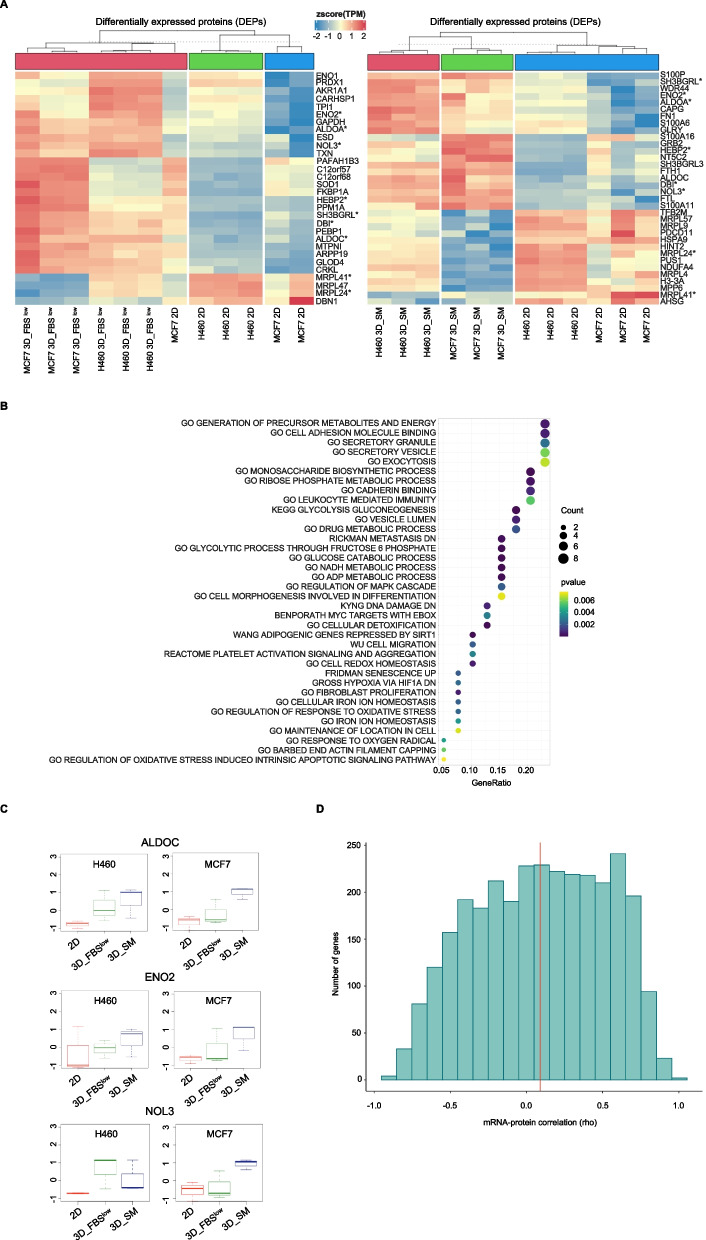
Proteomic analysis of H460 and MCF7 cell lines grown in 2D and 3D culture conditions. **A** Heatmap of 3DEPs in 3D *vs* 2D conditions of both cell lines. Common DEPs in all 3D vs 2D conditions are labeled with (*). Color intensity is proportional to the magnitude of changes. Relative expression levels are shown in red (upregulation) and blue (downregulation). **B** KEGG pathway analysis of DEPs in all 3D vs 2D conditions of both cell lines. The dot size denotes the number of DEPs, while colors correspond to the adjusted *p*-value range. **C** Dot plots showing ALDOC, ENO2, and NOL3 protein levels of H460 and MCF7 cell lines in 3D *vs* 2D conditions. **D** The distribution of gene-wise mRNA-protein correlations computed as Spearman’s Rho (x-axis). A histogram of 20 bins is shown with height of each bar proportional to the number of genes in each bin. The median correlation is depicted by a red vertical line

Once identified the gene expression signature associated with 3D tumor spheroid growth, we analyzed the proteomic profiles of H460, and MCF7 3D tumor spheroids grown either in SM or in FBS^low^ conditions and compared them to their relative 2D cultures. By using an absolute log_2_ |FC|> 1 and a *p-*value < 0.01, we identified a total of 534 DEPs in H460 3D_SM *vs* H460 2D, n = 413 DEPs in H460 3D_FBS^low^ *vs* H460 2D, n = 216 DEPs in MCF7 3D_SM *vs* MCF7 2D, and n = 222 DEPs in MCF7 3D FBS^low^ *vs* MCF7 2D (Table S5, in Additional file 5). Among these, 2 proteins (MRPL41 and MRPL24) were down regulated while 7 proteins (ALDOA, ALDOC, NOL3, ENO2, SH3BGRL, DBI, HEBP2) were up regulated in both H460 and MCF7 3D *vs* 2D conditions (Fig. 3A). Both the commonly down regulated proteins MRPL41 and MRPL24 are component of mitochondrial ribosomes (mitoribosomes) large 39S subunit and are involved in the synthesis of mitochondrial electrons transport chain (ETC) components [75, 76]. Among the commonly up regulated proteins, as already discussed above, the two isoenzymes ALDOA and ALDOC as well as ENO2 are glycolytic enzymes, NOL3 acts as apoptosis repressor, often in response to hypoxia, by inhibiting the release of cytochrome c from mitochondria [77], the Acyl-coA-binding protein DBI is a lipogenic factor that regulates fatty acids metabolism [78], the heme binding protein 2 (HEBP2) is involved in heme metabolism but it also enhances the outer and inner mitochondrial membrane permeabilization, especially under oxidative stress conditions [79]. The SH3 Domain Binding Glutamate Rich Protein Like (SH3BGRL) is located within the extracellular vesicles and as a scaffold protein it mediates many protein–protein interactions; however, its role in cancer is still largely undefined [80]. In agreement, KEGG enrichment analysis revealed that the common DEPs mainly affected metabolic and bioenergetic processes (i.e., *GO Generation of precursor metabolites and energy, GO Monosaccharide biosynthetic process, GO Ribose phosphate metabolic process, KEGG Glycolysis and gluconeogenesis*), exocytosis, cell adhesion processes (i.e., *GO Cell adhesion molecule binding, GO Cadherin binding*) and cellular response to oxidative stress (i.e., *GO Cell redox homesostasis*, *GO regulation of response to oxidative stress*) (Fig. 3B). Interestingly, when RNAseq and proteomic data were intersected, ALDOC, ENO2, and NOL3 emerged as significantly up regulated with a log_2_|FC|> 1 and *p-*value < 0.05 in all 3D *vs* 2D culture conditions both at gene and protein levels (Fig. 3C). Furthermore, we employed a novel bioinformatic protocol, able to jointly analyze transcriptomics and proteomics data, to gain additional insight on the relationship among the two omic profiles [45]. The analysis shows how mRNA/protein correlation levels span from 0.3 to 0.5 in different samples, with the H460_2D ranking at the top (Fig. S1, in additional file 6). These values are what expected in literature analysis, showing that only the subgroup of highly expressed genes show a strong correlation with protein levels [81]. Interestingly, a different overview of correlation levels in gene clusters show how clusters differentiate from small hyper-concordant groups (rho > 0.8%) to non-concordant outliers (rho < 0.2%) (Fig. 3D). It is safe to assume that the non-concordant outliers are more influenced in post-transcriptional modifications.

Collectively, proteomic data confirmed that cancer cells reprogram their glucose metabolic to adapt, and thus to survive, to the altered oxygen homeostasis caused by cellular reorganization of within 3D tumor spheroids and that this is independent from both cell type and nutrient availability.

### Metabolic profiling of H460 and MCF7 tumor spheroids indicate a shift toward a more pronounced glycolitic phenotype regardless of the cell culture conditions

**Fig. 4:**
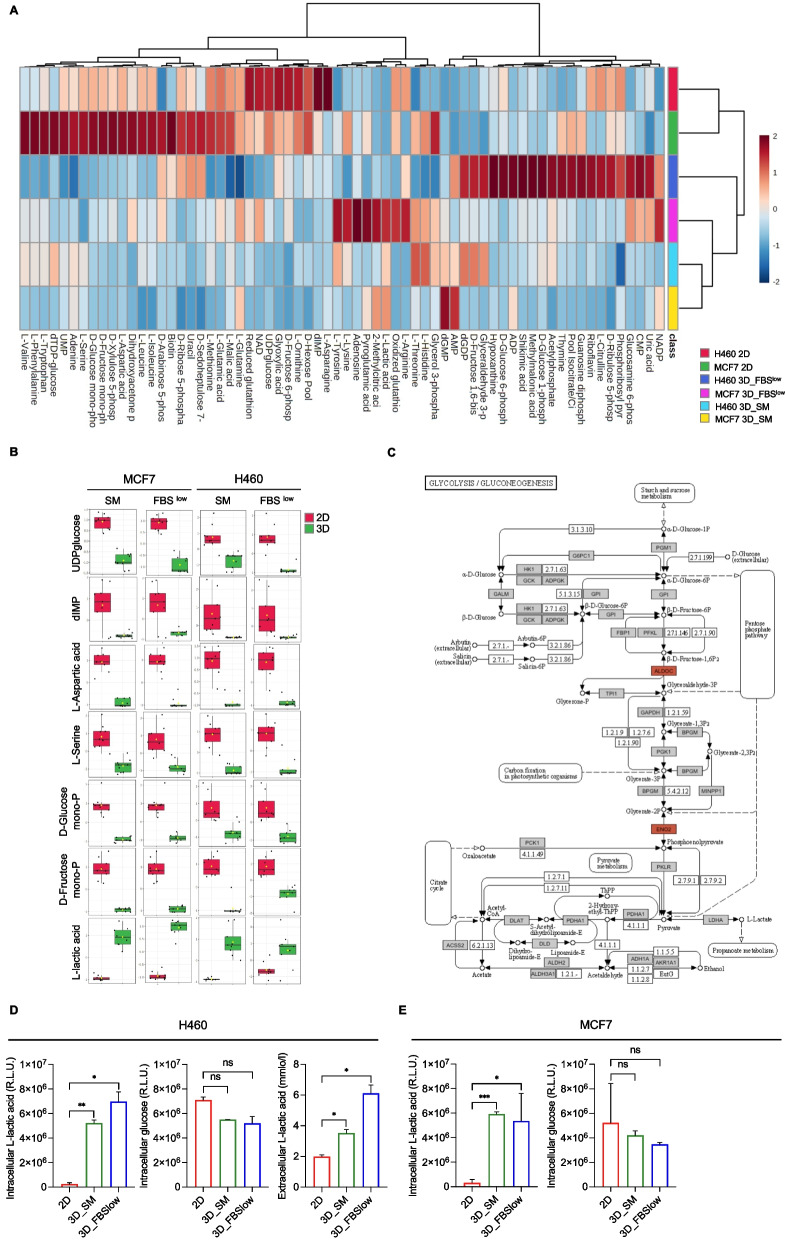
Metabolomic analysis of H460 and MCF7 cell lines grown in 2D and 3D culture conditions. **A** Heatmap of 66 significantly altered metabolites in H460 and MCF7 cell lines in 2D *vs* 3D conditions. **B** Dot plots showing the 7 metabolites with the same trend of variation in all 3D *vs* 2D cultures of both cell lines. **C** KEGG pathway enrichment analysis of glycolysis/gluconeogenesis, showing in red ALDOC and ENO2 upregulation at both gene and protein levels. **D**-**E** Intracellular glucose and L-lactic acid amounts measured by luminometric assays and reported as relative light units (R.L.U.); quantification of L-lactic acid within the culture media (extracellular) performed by emogas analysis and expressed as mmol/l in H460 2D, H460 3D_SM, H460 3D_ FBS^low^, MCF7 2D, MCF7 3D_SM, and MCF7 3D_ FBS^low^. All the experiments were carried out in triplicate and results are presented as mean ± SD. *p-*value: * < 0.05, ** < 0.01, *** < 0.001. ns: not significant

Prompted by the information arising from RNAseq and proteomic analysis, we decided to investigate the metabolic shift associated with changes in nutrient availability in non-adherent conditions. To this aim, we performed targeted polar metabolomic profiling of H460 and MCF7 cells grown as 2D, as well as 3D_SM and 3D_FBS^low^ tumor spheroids. Collectively, the LC–MS platform enabled us to detect 80 metabolites (Table S6, in Additional file 7). A total of 66 metabolites were found significantly altered among the three cell culture conditions (2D, 3D_SM and 3D_FBS^low^) with log_2_ |FC|> 1 and a *p-*value < 0.01. We observed that, as for the transcriptomic and proteomic profiles, the intracellular metabolomic profiles of H460 and MCF7 cells grown as 2D cultures were substantially different, as attested by the net clustering of samples shown in Fig. 4A. According to the literature, lung and breast cancer cells have different inherited metabo-phenotypes (metabotypes) and dependencies caused by the genetic background, the oncogenic evolution, and the interaction with the cellular niche [82]. H460 are primarily glycolytic cells [83]; MCF7, instead, are the most oxidative among the breast cancer cells, and overall display high flexibility in the substrate-driven ATP production [84]. In this regard, our data show that both H460 and MCF7 in 2D culture conditions consume glucose; however, the higher ratio isocitrate/citrate in MCF7 compared to H460 suggests a higher mitochondrial functionality in the breast cancer cell line than in the lung cancer cell line. In addition, as suggested by the higher amount of Ribose-5P, Xylulose-5P and Sedoheptulose-7P, MCF7 cells seem to promote anabolism through PPP for nucleotide synthesis, synthesis of serine and glycerol-3-P (Fig. 4A).

The amount of intracellular polar metabolites significantly diverged along the transition from 2 to 3D models regardless of the cell type. Overall, we identified 66 altered metabolites; among these, 7 showed the same trend of variation in all 3D *vs* 2D cultures: D-Glucose monophosphate, D-Fructose monophosphate, D-hexose pool, UDP glucose, dIMP, L-Aspartic Acid and L-Serine were significantly down-regulated in 3D *vs* 2D while L-lactic acid was the only metabolite up-regulated in 3D H460 and MCF7 compared to their relative adherent cells (Fig. 4A-B). It is important to note that, in FBS^low^ culture condition, MCF7 produced a higher amount of L-lactic acid compared to H460 cells, thus further suggesting the occurrence of a significant shift toward a glycolytic phenotype in the breast cancer cell line compared to the LUAD cell line which instead appeared more glycolytic already in 2D conditions. The increased intracellular ratio L-lactic acid/Glucose monophosphate and D-Fructose monophosphate in all 3D tumor spheroids compared to their relative 2D cultures well agreed with the up-regulation of the glycolytic enzymes ALDOC and ENO2, at both gene and protein levels (Fig. 4C; Fig. S2 in additional file 8). To confirm the shift toward a more pronounced glycolytic phenotype suggested by the metabolomic analysis, we quantified both intracellular glucose and L-lactic acid in 2D and 3D conditions by using specific luminometric assays. Results reported in Fig. 4D-E show a significant intracellular accumulation of L-lactic acid in all 3D conditions (*p-value < 0.05, **p-value < 0.01, ***p-value < 0.001) accompanied by a slight reduction in glucose (ns = not significant). In line with the increase of intracellular L-lactic acid levels, the analysis of L-lactic acid within the culture media (extracellular L-lactic acid) showed a significant release of this metabolite in H460 and MCF7 3D spheroids compared to their relative 2D counterparts (*p-value < 0.05) (Fig. 4D-E).

### MCF7 show a greater ability to generate 3D tumor spheroids in nutrient-restricted culture conditions compared to H460 cells

**Fig. 5:**
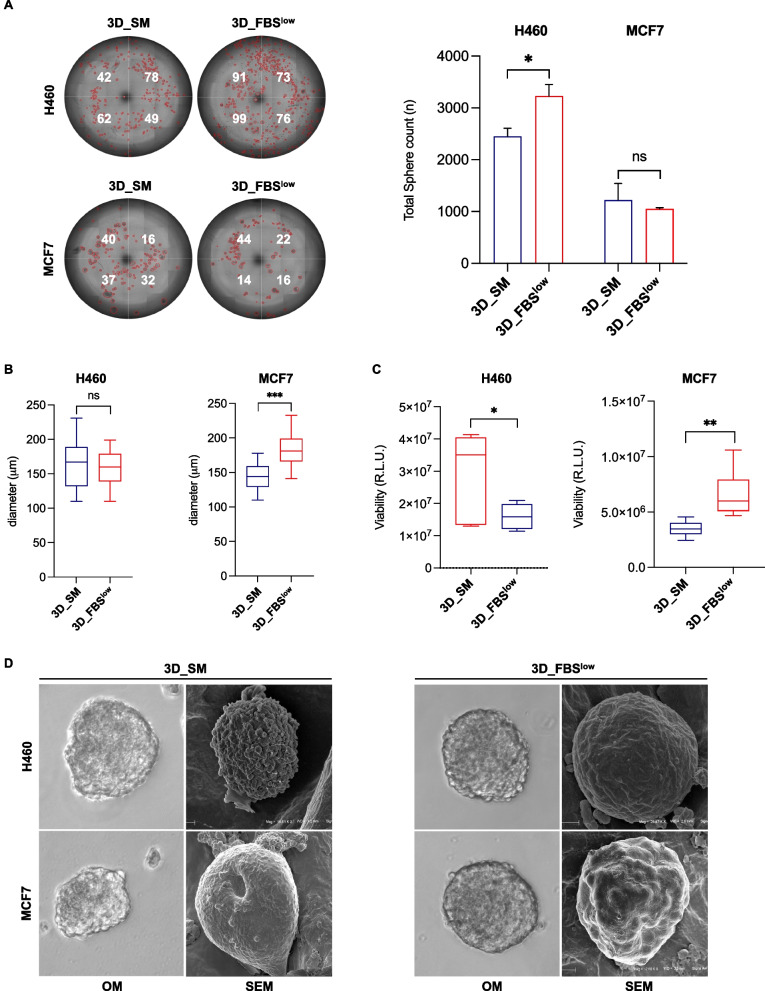
Analysis of morphology and growth rate of H460- and MCF7-derived tumor spheroids. **A** Representative images and relative histograms of H460 3D_SM, H460 3D_ FBS^low^, MCF7 3D_SM, and MCF7 3D_FBS^low^ tumor spheroids morphology, count and **B** diameter. **C** Cell viability of H460 3D_SM, H460 3D_FBS^low^, MCF7 3D_SM, and MCF7 3D_FBS^low^ assessed by Cell titer-Glo 3D assay and expressed as relative light unit (RLU). **D** Representative images of H460 3D_SM, H460 3D_FBS^low^, MCF7 3D_SM, and MCF7 3D_FBS^low^ tumor spheroids obtained by SEM. All the experiments were carried out in triplicate and results are presented as mean ± SD. *p*-value: * < 0.05, ** < 0.01. *** < 0.001. ns: not significant

Gene and protein expression reorganization associated with 3D cell culture drive morphological and functional changes, such as proliferation rate and drug resistance [85]. Here, we observed that nutrient restriction had different effects on both tumor spheroids size and number depending on the cell type analyzed. Indeed, the FBS^low^ culture condition caused an increase of H460 tumor spheroids number compared to SM (3230 ± 221 (FBS^low^)) *vs* (2450 ± 158 (SM)) (*p*-value < 0.05) without significantly affecting their diameter (163.9 ± 30.8 (SM) vs 158.51 ± 25.55 (FBS^low^), ns). The number of tumor spheroids deriving from MCF7 cells was instead apparently unaffected by the different culture conditions (1050 ± 24 (FBS^low^) *vs* 1223 ± 320 (SM), ns), but they appeared increased in size when grown in the FBS^low^ culture medium (156.99 ± 26.59 (SM) vs 180.02 ± 22.43 (FBS^low^), *p*-value < 10^–7^) (Fig. 5A-B). Cell viability assay highlighted that while H460 cells suffered from nutrient-restricted culture medium (FBS^low^) MCF7 cells, grown in the same culture condition, showed an enhanced cell viability (Fig. 5C). This difference can be attributed to the previously mentioned higher inherited metabolic plasticity of MCF7 cells, which therefore result more adaptable to nutrient restrictions and, overall, less dependent on glucose to produce ATP.

Finally, Scanning Electron Microscopy (SEM) analysis revealed that the plasma membrane ultrastructural features of 3D spheroids appeared morphologically distinguishable depending on culture conditions. Notably, both H460- and MCF7-derived spheroids cultured in SM showed intense plasma membrane blebbing, indicating high membrane dynamics with respect to FBS^low^ cultured counterpart. Since this activity can be related to microvesicles formation this aspect deserves further investigations. Moreover, H460-derived tumor spheroids grown in FBS^low^ appeared more compact, provided with a marked roundness, suggesting a different junctional behaviour of SM and FBS^low^ cultured samples (Fig. 5D). Collectively, these results suggest that both H460 and MCF7 cells survive to harsh nutrient culture conditions and generate tumor spheroids that appear more compact; besides, MCF7 appear favoured in terms of spheroids size and growth rate possibly because of their inherited metabolic plasticity.

### Suppression of ALDOC and ENO2 restrains 3D tumor spheroids growth of H460 and MCF7 cells

**Fig. 6:**
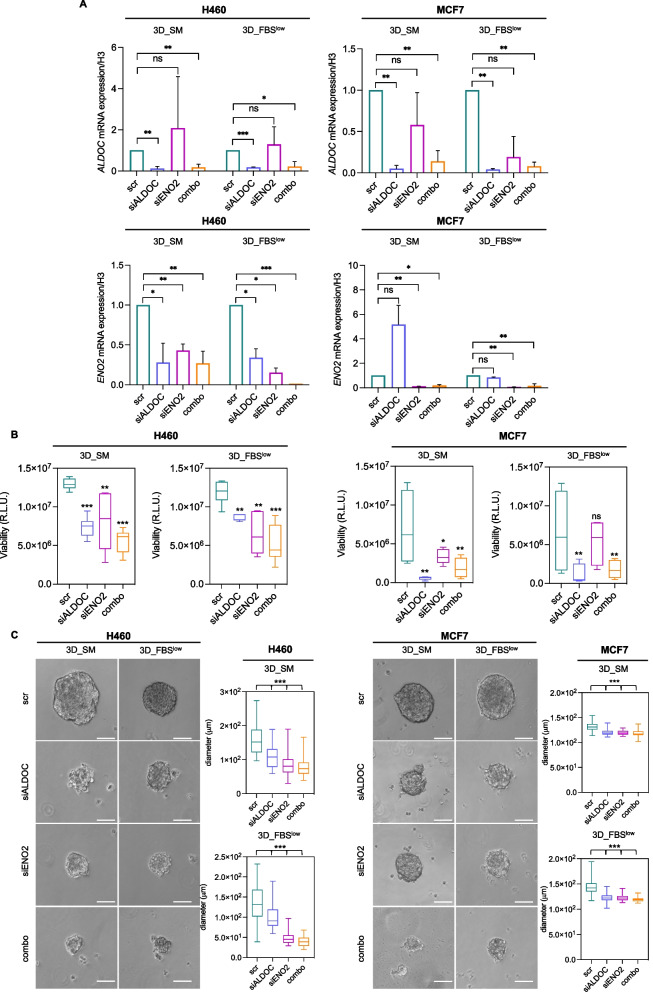
*ALDOC* and *ENO2* knock down reduces the sphere-forming ability of H460 and MCF7 cells. **A** qRT-PCR analyses of *ALDOC* and *ENO2* in H460 3D_SM, H460 3D_ FBS^low^, MCF7 3D_SM, and MCF7 3D_ FBS^low^ upon *ALDOC* and *ENO2* silencing alone or in combination. **B** Cell viability of H460 3D_SM, H460 3D_FBS^low^, MCF7 3D_SM, and MCF7 3D_FBS^low^ upon *ALDOC* and *ENO2* silencing alone or in combination assessed by Cell titer-Glo 3D assay and expressed as relative light unit (R.L.U). **C** Representative images and relative histograms of tumor spheroids morphology and diameter of H460 3D_SM, H460 3D_FBS^low^, MCF7 3D_SM, and MCF7 3D_FBS^low^ upon *ALDOC* and *ENO2* silencing alone or in combination. All the experiments were carried out in triplicate and results are presented as mean ± SD. *p-*value: * < 0.05, ** < 0.01, *** < 0.001. ns: not significant

To confirm the role of ALDOC and ENO2 in H460 and MCF7 tumor spheroids growth we performed the transient knock down of both enzymes. Data reported in Fig. 6A show that single knock down of *ALDOC* and *ENO2* led to the evident reduction of each gene, which was more prominent when both genes were silenced together. Furthermore, we observed that *ENO2* silencing did not affect *ALDOC* gene expression levels; conversely, *ALDOC* knock down significantly reduced *ENO2* only in H460 cells regardless of the culture media conditions.

**Fig. 7:**
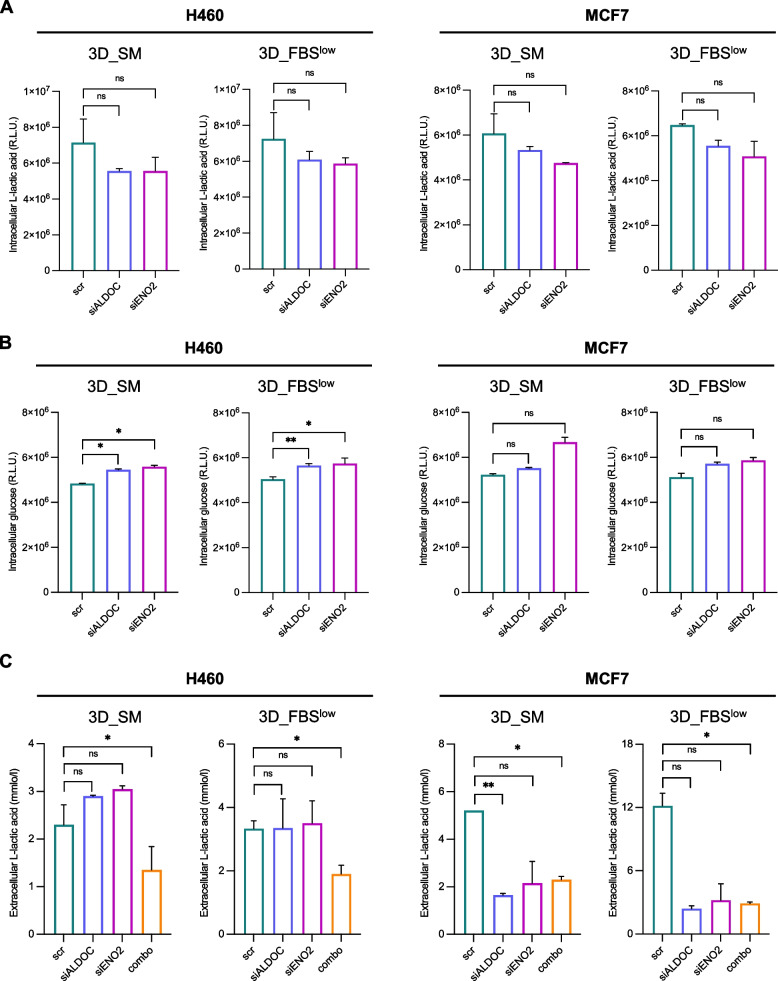
*ALDOC* and *ENO2* knock down impairs glucose and L-lactic acid amounts in H460 and MCF7 3D tumor spheroids. **A-B** Intracellular glucose and L-lactic acid amounts measured by luminometric assays and reported as relative light units (R.L.U.) in H460 3D_SM, H460 3D_ FBS^low^, MCF7 3D_SM and MCF7 3D_ FBS^low^ upon *ALDOC* and *ENO2* silencing alone. **C** Quantification of L-lactic acid within the culture media (extracellular) performed by emogas analysis and expressed as mmol/l in H460 3D_SM, H460 3D_ FBS^low^, MCF7 3D_SM and MCF7 3D_ FBS^low^ upon *ALDOC* and *ENO2* silencing alone or in combination. All the experiments were carried out in triplicate and results are presented as mean ± SD. *p-*value: * < 0.05, ** < 0.01, *** < 0.001. ns: not significant

Next, we found that *ALDOC* and *ENO2* knock down, either as a single entity or in combination, attenuated the spheroids forming ability in both cell lines regardless of the culture media conditions as shown by the markedly reduced cell viability (Fig. 6B) and tumor spheroids size (see images and relative histograms in Fig. 6C) (*p-value < 0.05, **p-value < 0.01, ***p-value < 0.001). Based on these results, we wondered whether H460 and MCF7 growth in non-adherent conditions was dependent on ALDOC- and ENO2-mediated glucose metabolism. To this, we assessed the effects of transient knock down of *ALDOC* and *ENO2* on intracellular glucose and L-lactic acid amounts as well as on L-lactic acid release within culture media. As shown in Fig. 7A-B, knock down of *ALDOC* and *ENO2* alone caused, although without reaching the statistical significance, a reduction of both intracellular and extracellular L-lactic acid as well as a slight increase in glucose amounts in both cell lines and culture media. The only exception was represented by the intracellular glucose amounts which appeared significantly increased upon ALDOC or ENO2 silencing alone in H460 cells regardless of the cell culture conditions (*p-value < 0.05, **p-value < 0.01). Silencing of both enzymes together, instead, led to the marked reduction of L-lactic acid production and release (*p-value < 0.05) in both the cell lines (Fig. 7C). Collectively, the biological effects observed upon ALDOC and/or ENO2 transient knock down are reported in Fig. S3 (additional file 9).

It is important to note that data shown in Fig. 6 and 7 are the results of ALDOC and ENO2 silencing performed by using a specific siRNA for each gene. Two additional siRNAs (siALDOC #2/3; siENO2 #2/3) were also used to knock down each gene to exclude any off-targets effects as shown in Fig. S4 and S5 in additional files 10–11. Overall, these results indicate that the loss of either ALDOC or ENO2 significantly impairs the ability of both H460 and MCF7 to in terms of spheroids viability and size, regardless of the culture media conditions. This effect appears exacerbated when tumor spheroids are, in parallel, deprived of both enzymes. Notably, the transient knock down of each gene leads to the perturbation of the glycolytic flux, although without reaching the statistical significance in most of the 3D conditions. Among the two cell types, H460-derived tumor spheroids seem more affected by the single knock down of *ALDOC*, which alone causes the significant down regulation of *ENO2* and the significant accumulation of intracellular glucose amount. We hypothesize that this could be explained, once again, by the already discussed primarily glycolytic phenotype of H460 cells [83]. The glycolytic flux results significantly perturbed in both cell types upon then loss of both enzymes, as demonstrated by the significant reduction of L-lactic acid production and release.

**Fig. 8:**
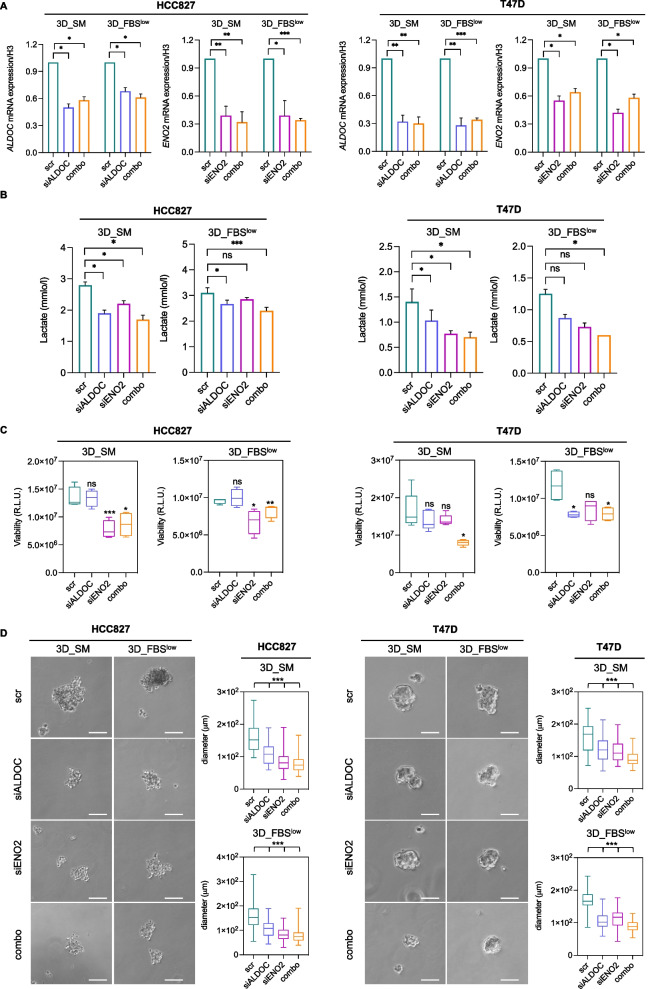
*ALDOC* and *ENO2* knock down reduces the sphere-forming ability of HCC827 and T47D cells. **A** qRT-PCR analyses of *ALDOC* and *ENO2* in HCC827 3D_SM, HCC827 3D_FBS^low^, T47D 3D_SM, and T47D 3D_FBS^low^ upon *ALDOC* and *ENO2* silencing alone or in combination. **B** L-lactic acid production assessed in HCC827 3D_SM, HCC827 3D_FBS^low^, T47D 3D_SM, and T47D 3D_FBS^low^ upon *ALDOC* and *ENO2* silencing alone or in combination.** C** Cell viability of HCC827 3D_SM, HCC827 3D_FBS^low^, T47D 3D_SM, and T47D 3D_FBS^low^ upon *ALDOC* and *ENO2* silencing alone or in combination assessed by Cell titer-Glo 3D assay and expressed as relative light unit (RLU). **D** Representative images and relative histograms of tumor spheroids morphology and diameter of HCC827 3D_SM, HCC827 3D_FBS^low^, T47D 3D_SM, and T47D 3D_FBS^low^ upon *ALDOC* and *ENO2* silencing alone or in combination. All the experiments were carried out in triplicate and results are presented as mean ± SD. *p-*value: * < 0.05, ** < 0.01, *** < 0.001. ns: not significant

These results were further confirmed in additional LUAD (HCC827) and breast cancer (T47D) cell lines. Indeed, as shown in Fig. 8, the combined knock down of the two glycolytic enzymes significantly impaired lactate production and hampered the growth of HCC827 and T47D cells as 3D tumor spheroid in non-adherent conditions.

## Discussion

Cell adaptation, selection, and evolution are key processes along all the steps of tumor initiation and progression, including the propensity of cancer cells to leave the primary site, migrate and establish metastases [86]. To leave the primary tumor, cancer cells adopt drastic transcriptional and metabolic changes that jointly initiate the invasion-metastatic cascade and, thus, allow tumor cells to detach from the ECM, adopt an EMT phenotype and disseminate from primary lesions into the blood or the lymphatic vessels [87]. Then, circulating tumor cells adopt anti-anoikis, or anchorage-independent survival mechanisms, to further adapt to the severe environmental stress imposed by separation from the ECM [88–90]. According to the literature, oxygen, energy metabolism and redox homeostasis are three inextricably linked factors among which cancer cells need to strike a balance to survive under detached conditions [91]. Only a small subpopulation of persisting cancer cells leaving primary tumors are able to maintain an optimal balance between the competing interests arising from three factors and, thus, to successfully reach metastatic secondary sites.

In addition to an “imprinted” predisposition and/or the acquisition of random mutational hits, persisting cancer cells show a remarkable plasticity against the metabolic requirements imposed by the different microenvironments during the different steps of the metastatic cascade. Such metabolic reprogramming can be controlled both through the transcriptional and post-transcriptionally regulation of specific enzymes or through metabolite availability [92]. In this regard, it has been demonstrated that lactate and pyruvate metabolism promote the switch from a proliferative to a migrating metastatic cell phenotype through the modulation of different signalling pathways and global gene expression programmes. In breast cancer cells, the reduction of the oxidative metabolism in favor of glycolytic energy production leads to the accumulation of acetyl-CoA and the consequent acetylation of the transcription factor Smad2, which is a well-known inducer of the mesenchymal genes patterns [93]. In agreement, both metastatic lung cancer cell lines and metastases isolated from lung cancer mouse models show downregulated gene expression of proteins belonging to the ETC [94]. In breast cancer cells, lactate dehydrogenase (LDH) has been found to be phosphorylated and thus activated by HER2 and SRC, and that the inhibition of such phosphorylation is associated with decreased invasiveness [95]. Once in blood circulation, lactate and pyruvate also contribute to the resistance to the hypoxia-mediated ROS accumulation within cell clusters through the stabilization of HIF1α protein [96]. Alternatively, glutamine metabolism is also involved in tumor invasion. To make an example, the overexpression of glutaminase 1 (GLS1), which catabolizes the conversion from glutamine to glutamate, is required for colorectal cancer cells migration and lymphnode metastasis [97]. In agreement, glutamate dehydrogenase (GDH), which converts glutamate to α-ketoglutarate, has been identified as a prognostic marker of colorectal cancer metastasis [98]. Alterations in lipid metabolism is also intimately linked to tumor progression. For instance, the increase in monounsaturated fatty acids, generated by the activity of SCDs, the rate-limiting enzymes in the formation of monounsaturated fatty acids, is associated with the acquisition of cancer stem cells (CSCs)-like features in ovarian and lung cancer cells lines [99–101]. In agreement, the increased activity of SCD1 has been found to promote YAP/TAZ signalling pathway thus enhancing melanoma CSCs aggressiveness [102]. Similarly, the activation of the mevalonate pathway, responsible for cholesterol synthesis, confers stem cell traits to breast cancer cells. In agreement, the inhibition of HMG-CoA reductase, the rate-limiting enzyme of the mevalonate cascade, resulted effective against breast cancer stem cells [103].

In this study, by using a well-integrated multi-omics approach, we demonstrate that the ability of H460 LUAD and MCF7 breast cancer cells to grow in non-adherent condition and to generate 3D tumor spheroids both in glucose-rich (3D_SM) and glucose-deprived (3D_FBS^low^) culture media is associated with a consistent modulation of genes and proteins mainly involved in metabolic reprogramming towards an enhanced glycolytic phenotype most likely induced by a hypoxic condition. Indeed, we found that all the transitions from 2 to 3D cultures, regardless of the cancer cell type and cell culture conditions, are accompanied by the significant up-regulation of genes encoding for 6 out of 10 glycolytic enzymes: *HK2* and *ALDOC* belonging to the energy-requiring phase and *GAPDH, PGK1, ENO2,* and *PKM* belonging to the energy-releasing phase [104]. In agreement with transcriptomic data, our metabolomic analyses highlighted a significant consumption of glucose and a corresponding increase in lactate production in all 3D tumor spheroids compared to their relative 2D parental cells, even within glucose-restricted culture conditions. If on the one hand, these results leant towards a mandatory role of the glycolytic cascade for the maintenance of cancer cell survival under detached-culture conditions, on the other hand raised the question of how 3D tumor spheroids enhanced their glycolytic phenotype in glucose-restricted conditions.

Glucose metabolism is one of the major metabolic pathways essential for tumor growth [105]. According to the “Warburg effect” concept, tumor cells enhance glycolytic cascade and the LDH- mediated lactate production both under hypoxic and normoxic conditions [106]. Warburg effect allows tumor cells to gain survival advantages in two ways: one is to increase carbon sources, which are used to synthesize proteins, lipids, and nucleic acids to meet the needs of tumor growth; the other one is to turn off the aerobic respiration to suppress ROS generation, thereby preventing cell death [107]. In particular under hypoxia conditions, cancer cells tend to enhance lactate production by enhancing the expression of glycolytic enzymes and lactate dehydrogenase (LDH) [108]. In this regard, our results show that in association with the increase in lactate production all the 2D to 3D transitions are accompanied by the overexpression of *LDH*, although without reaching the statistical significance (Table S1). The overproduction of lactate represents a well-documented benefit for cancer cells through the acidification of the tumor microenvironment (TME), VEGF-mediated angiogenesis, increase of cancer cell motility and self-renewal of cancer stem cells (CSCs) [108]. As such, lactate is positively associated with tumor metastasis and recurrence [109, 110].

During hypoxic conditions, cancer cells also use other mechanisms to foster the conversion of pyruvate to lactate. Among these, one is the inhibition of pyruvate entry into the TCA cycle through the PGK1-mediated phosphorylation of pyruvate dehydrogenase kinase 1 (PDK1), which in turn inhibits the pyruvate dehydrogenase complex (PDC) [111]. Interestingly, PGK1 is one of the glycolytic genes up regulated in all 3D *vs* 2D conditions. Gene expression analysis of 3D glioblastoma spheroids has shown increased expression of pyruvate dehydrogenase kinase 4 (PDK4) involved in the suppression of mitochondrial activity. In agreement, MYC, involved in mitochondrial energy production, was found down-regulated and the evaluation of TCA cycle metabolic products showed decreases in the levels of succinate, fumarate, and malate [112]. Interestingly, the same changes have been reported in metabolomic analysis of ovarian cancer cell spheroids [113].

The induction of mitochondrial autophagy (mitophagy), in concert with inhibition of mitochondrial biogenesis, represents critical adaptive mechanism to maintain oxygen homeostasis and prevent mitochondrial ROS accumulation under hypoxic conditions [114, 115]. Importantly, our transcriptomic and proteomic data show a significant alteration of genes and proteins involved both in mitochondria biogenesis and clearance. All the transitions from 2 to 3D conditions, in fact, were characterized by i) the significant down regulation of MRPL41 and MRPL24 components of mitoribosomes involved in the synthesis of mitochondrial electrons transport chain (ETC) components [75], ii) the significant up-regulation of BINP3 and BNIP3L that are targets of HIF1 and are necessary for mitophagy [63]. BINP3/BINP3L are involved in mitochondrial quality control: in response to mitochondrial damage, they participate to the degradation of damaged proteins inside mitochondria and in the opening of the pores within the mitochondrial double membrane in order to mediate the translocation of the lysosomal proteins from the cytoplasm to the mitochondrial matrix.

As last adaptive mechanism, HIF-1 reprograms tumor metabolism by increasing glycogen reserves under hypoxia [116]. According to the literature, a decrease in pO2 acts as an “alarm” that prepares cancer cells to face subsequent nutrient depletion through the induction of glycogen storage. In this regard, our findings demonstrate that the mRNA levels of the first enzyme of the glycogenesis PGM1 were increased in all 3D vs 2D conditions, regardless of the culture media. In agreement, metabolomic analysis shows a significant decrease in the intracellular level of uridine diphosphate glucose (UDP) which is the first substrate for glycogen synthesis.

In addition to glucose, recent studies suggest that fructose can be preferentially metabolized by cancer cells under low oxygen conditions through an alternative catabolic pathway known as fructolysis [117]. During fructolysis fructose is first converted to fructose 1-phosphate by fructokinase and then converted to DHAP and G3P specifically by the aldolase isoforms ALDOB and ALDOC. In this regard, our results show that all 2D to 3D transitions were associated with the significant up-regulation of ALDOC isoenzyme at both gene and protein level concomitant with the significant reduction of the intracellular levels of fructose-monophosphate. Stemming from these observations, we could also hypothesize that, in non-adherent conditions, certain cancer cells, and above all those deprived of glucose, might become fructose-dependent. According to the literature, fructolysis show several advantages for cancer cells compared to glycolysis. First, fructose can be quickly catalyzed because fewer enzymes are involved in this process than in glycolysis. Fructolysis fuels glycolysis thus leading to a further increase in lactate production [118]. Indeed, since fructokinase activation sequesters a phosphate from ATP, the consequent ATP and phosphate depletion enhances glycolysis by activating the glycolytic enzymes PFK and PK [119]. Furthermore, G3P generated by ALDOB and ALDOC during fructolysis enters to the glycolytic pathway distal to PFK [53]. The rapid reduction of phosphate caused by the activation of fructokinase has been shown to activate the AMP deaminase (AMPD), which cleaves AMP to IMP [117]. The latter is used to generate acid uric, which in turn causes mitochondrial ROS accumulation [120]. In line with these data, our results show that 3D tumor spheroids were characterized by the up-regulation of AMPD3 isoform and a significant reduction of IMP intracellular levels. AMPD3 is involved in the activation of AMPK, which is largely recognized as an early energy sensor activated by glucose deprivation and responsible of the activation of alternative catabolic pathways to generate ATP [121].

Together with ALDOC, also ENO2 was found up-regulated Interestingly, both ALDOC and ENO2 are neuro-specific isoforms of the relative enzymes mainly expressed in normal neuronal tissues [53, 54]. As such, their overexpression in 3D tumor spheroids derived from LUAD and breast cancer cell lines was somehow unexpected; however, it could be suggestive of a broad neuronal-specific gene expression reprogramming of cancer cells during detachment from the ECM and 3D tumor growth. Although still poorly defined, the literature suggests that both isoenzymes exert non-canonical “moonlighting” functions in carcinogenesis [122, 123]. Under hypoxia, HIF1a binds to the hypoxia- responsive element (HRE) on the promoter region of ALDOC, thus causing metabolic reprogramming or aberration of glycolysis to promote glioblastoma and ovarian cancer [124]. In 2022 Maruyama R et al. demonstrated that ALDOC is overexpressed in 3D tumor spheroids derived from colorectal cancer (CRC) cell lines and that its overexpression in CRC patients correlated with metastasis and poor prognosis [125]. ENO2 can function as on oncogene, either in neuronal malignancies or in other cancer types, such as lung, breast, and prostate cancer [126–128]. Recent evidence was provided that the C-term domain of ENO2, which is not necessary for metabolic activity, activates the MAPK/ERK signaling pathway and thus promotes proliferation and migration of BRAV V600E-mutated CRC cells [129]. In this regard, in our study, we demonstrate that the combined knockdown of ALDOC and ENO2 significantly reduced lactate production and consequently attenuated the sphere-forming ability of both LUAD and breast cancer cell lines both in nutrient-rich and nutrient-restricted conditions.

Finally, the integration of transcriptomic and proteomic data highlighted that NOL3 was up regulated in all 3D vs 2D conditions both at gene and protein levels. NOL3 functions as a suppressor of both intrinsic and extrinsic apoptosis through several mechanisms, including the blockage of death-inducing signaling complex (DISC) assembly, the limitation of caspase-8 for DISC-mediated activation, and the inactivation of pro-apoptotic BAX [130].

## Conclusions

Overall, the present work shows that the integration of transcriptomic, proteomic, and metabolomic analyses is a powerful approach to unveiling in-depth global adaptive cellular responses and the interconnection of regulatory circuits involved in the ability of cancer cells to survive in non-adherent conditions. Indeed, our findings reveal that an extensive metabolic rewiring towards an increased glycolytic “metabotype” and an enhanced lactate production is mandatory to achieve a new homeostasis state that favors cancer cell survival in 3D culture conditions. This phenomenon is accompanied by multiple adaptive events of both transcriptional and translational machineries that merge to a hypoxic-mediated upregulation of anaerobic glycolytic cascade, maintenance of intracellular redox homeostasis, activation of autophagic and antiapoptotic pathways. Noteworthy, in all the transitions from 2 to 3D cultures, ALDOC and ENO2 glycolytic enzymes are upregulated both at transcriptional and translational levels and interfering with their activity is sufficient to repress lactate production and to reduce sphere-forming ability of both LUAD and breast cancer cell lines. This result suggests that ALDOC and ENO2 may represent new powerful targets to restrain 3D tumor spheroids generation of both lung and breast cancer cell lines cultured in different environmental nutrient availability.

## Supplementary Information

**Additional file 1: Table S1.** RNAseq data.

**Additional file 2: Table S2.** GO_Biological_Process.

**Additional file 3: Table S3.** GO_Molecular Function.

**Additional file 4: Table S4.** GO_Cellular_Component.

**Additional file 5: Table S5.** Proteomic data.

**Additional file 6: Figure S1.** Correlations between mRNA expression and protein abundance. Sample-wise mRNA-protein correlation computed as Spearman’s Rho (y-axis). Samples are ordered along the x-axis based on increasing correlation.

**Additional file 7.**

**Additional file 8: Figure S2.** ALDOC and ENO2 levels increase in all H460 and MCF7 3D conditions compared to their 2D relative counterparts. **A-B **qRT-PCR and Western Blot analyses of ALDOC and ENO2 in H460 2D, H460 3D_SM, H460 3D_FBS^low^, MCF7 2D, MCF7 3D_SM, and MCF7 3D_FBS^low^. All the experiments were carried out in triplicate and results are presented as mean ± SD. *p-*value: *<0.05.

**Additional file 9: Figure S3.** Schematic illustration of upstream and downstream events of *ALDOC* and/or *ENO2* perturbation.

**Additional file 10: Fig. S4. **ALDOC and ENO2 knock down with additional siRNAs confirms the reduction of sphere-forming ability of H460 and MCF7 cells. A qRT-PCR analyses of ALDOC and ENO2 in H460 3D_SM, H460 3D_FBS^low^, MCF7 3D_SM, and MCF7 3D_FBS^low^ upon ALDOC and ENO2 silencing with additional siRNAs. B Cell viability of H460 3D_SM, H460 3D_FBS^low^, T47D 3D_SM, and T47D 3D_FBS^low^ upon ALDOC and ENO2 silencing with additional siRNAs assessed by Cell titer-Glo 3D assay and expressed as relative light unit (R.L.U.). D Representative images and relative histograms of tumor spheroids morphology and diameter of H460 3D_SM, H460 3D_FBS^low^, MCF7 3D_SM, and MCF7 3D_FBS^low^ upon ALDOC and ENO2 silencing with additional siRNAs. All the experiments were carried out in triplicate and results are presented as mean ± SD. *p-*value: *<0.05, **<0.01, ***<0.001. ns: not significant.

**Additional file 11: Fig. S5.** ALDOC and ENO2 knock down with additional siRNAs also impairs glucose and L-lactic acid amounts in H460 and MCF7 3D tumor spheroids. A-B Intracellular glucose and L-lactic acid amounts measured by luminometric assays and reported as relative light units (R.L.U.) in H460 3D_ SM, H460 3D_FBS^low^, MCF7 3D_SM and MCF7 3D_FBS^low^ upon ALDOC and ENO2 silencing with additional siRNAs. C Quantification of L-lactic acid within the culture media (extracellular) performed by emogas analysis and expressed as mmol/1 in H460 3D_SM, H460 3D_ FBS^low^, MCF7 3D_SM and MCF7 3D_ FBS^low^ upon ALDOC and ENO2 silencing with additional siRNAs. All the experiments were carried out in triplicate and results are presented as mean = SD. ns: not significant.

## Abbreviations

LUAD: Lung adenocarcinoma
ALDOC: Aldolase C
ENO2: Enolase 2
ECM: Extracellular matrix
ATP: Diminishes adenosine triphosphate
ROS: Reactive oxygen species
FGF: Fibroblast growth factor
EGF: Epidermal growth factor
FBS: Fetal bovine serum
SM: Sphere medium
DEGs: Differential expressed genes
DEPs: Differential expressed proteins
RNAseq: RNA sequencing
PCA: Principal component analysis
GO: Gene ontology
DIA: Data-independent mode
HK2: Enzymes hexokinase 2
PKM: Pyruvate kinase muscle isozyme
PGK1: Phophoglycerate kinase 1
GAPDH: Glyceraldehyde 3-phosphate dehydrogenase
PGM1: Phosphoglucomutase 1
G3P: Gylceraldehydes-3-phosphate
DHAP: Dihydroxyacetone phosphate
HIF-1: Hypoxia-inducible factor-1
NDRG1: N-myc downstream-regulated gene-1
EMT: Epithelial to mesenchymal transition
HILPDA: Hypoxia-inducible and lipid droplet associated protein
RORA: Retinoic acid-receptor-related orphan receptor α superfamily
EV: Extracellular vescicles
AMPD3: Adenosine Monophosphate Deaminase 1
AMP: Adenosine monophosphate deaminase
IMP: Inosine monophosphate
AMPK: AMP-activated protein kinase
EFEMP2: EGF Containing Fibulin Extracellular Matrix Protein 2
SH3BGRL: SH3 Domain Binding Glutamate Rich Protein Like
SEM: Scanning Electron Microscopy; 1
LDH: Lactate dehydrogenase
TME: Tumor microenvironment
HRE: Hypoxia- response element
CRC: Colorectal cancer

## References

[1] Raimo D, di Raimo T, de Santis E, Coppola L, Rosario D’andrea M, Angelini F (2018). Circulating tumor cells and the metastatic process: the complexity of malignancy. J Cancer Metastasis Treat.

[2] Guan X (2015). Cancer metastases: challenges and opportunities. Acta Pharm Sin B.

[3] Hernández-Camarero P, López-Ruiz E, Marchal JA, Perán M (2021). Cancer: a mirrored room between tumor bulk and tumor microenvironment. J Exp Clin Cancer Res..

[4] Buchheit CL, Weigel KJ, Schafer ZT (2014). Cancer cell survival during detachment from the ECM: multiple barriers to tumour progression. Nat Rev Cancer..

[5] Adeshakin FO, Adeshakin AO, Afolabi LO, Yan D, Zhang G, Wan X (2021). Mechanisms for modulating anoikis resistance in cancer and the relevance of metabolic reprogramming. Front Oncol.

[6] Deng Z, Wang H, Liu J, Deng Y, Zhang N (2021). Comprehensive understanding of anchorage-independent survival and its implication in cancer metastasis. Cell Death Dis.

[7] Ascenzi F, de Vitis C, Maugeri-Saccà M, Napoli C, Ciliberto G, Mancini R (2021). SCD1, autophagy and cancer: implications for therapy. J Exp Clin Cancer Res..

[8] Yadav UP, Singh T, Kumar P, Sharma P, Kaur H, Sharma S (2020). Metabolic adaptations in cancer stem cells. Front Oncol.

[9] Wells A (2000). Tumor invasion: role of growth factor-induced cell motility. Adv Cancer Res..

[10] Ribatti D, Tamma R, Annese T (2020). Epithelial-mesenchymal transition in cancer: a historical overview. Transl Oncol..

[11] Fabricius HÅ, Starzonek S, Lange T (2021). The role of platelet cell surface P-Selectin for the direct platelet-tumor cell contact during metastasis formation in human tumors. Front Oncol.

[12] Gay LJ, Felding-Habermann B (2011). Contribution of platelets to tumor metastasis. Nat Rev Cancer.

[13] Hassan G, Ohara T, Afify SM, Kumon K, Zahra MH, Fu X (2022). Different pancreatic cancer microenvironments convert iPSCs into cancer stem cells exhibiting distinct plasticity with altered gene expression of metabolic pathways. J Exp Clin Cancer Res..

[14] Yi M, Li J, Chen S, Cai J, Ban Y, Peng Q (2018). Emerging role of lipid metabolism alterations in cancer stem cells. J Exp Clin Cancer Res..

[15] Feng C, Li Y, Li K, Lyu Y, Zhu W, Jiang H (2021). PFKFB4 is overexpressed in clear-cell renal cell carcinoma promoting pentose phosphate pathway that mediates Sunitinib resistance. J Exp Clin Cancer Res..

[16] Noto A, de Vitis C, Pisanu ME, Roscilli G, Ricci G, Catizone A (2017). Stearoyl-CoA-desaturase 1 regulates lung cancer stemness via stabilization and nuclear localization of YAP/TAZ. Oncogene..

[17] Lobello N, Biamonte F, Pisanu ME, Faniello MC, Jakopin Ž, Chiarella E (2016). Ferritin heavy chain is a negative regulator of ovarian cancer stem cell expansion and epithelial to mesenchymal transition. Oncotarget..

[18] Pisanu ME, Noto A, de Vitis C, Morrone S, Scognamiglio G, Botti G (2017). Blockade of Stearoyl-CoA-desaturase 1 activity reverts resistance to cisplatin in lung cancer stem cells. Cancer Lett..

[19] Ewels PA, Peltzer A, Fillinger S, Patel H, Alneberg J, Wilm A (2020). The nf-core framework for community-curated bioinformatics pipelines. Nat Biotechnol.

[20] Guglielmelli P, Biamonte F, Spolverini A, Pieri L, Isgrò A, Antonioli E (2010). Frequency and clinical correlates of JAK2 46/1 (GGCC) haplotype in primary myelofibrosis. Leukemia..

[21] R: a language and environment for statistical computing [Internet]. [cited 2022 Aug 1]. Available from: https://www.gbif.org/en/tool/81287/r-a-language-and-environment-for-statistical-computing

[22] R: The R Project for Statistical Computing [Internet]. [cited 2022 Aug 1]. Available from: https://www.r-project.org/

[23] | bioRxiv [Internet]. [cited 2022 Aug 1]. Available from: https://www.biorxiv.org/node/

[24] Prestagiacomo LE, Gabriele C, Morelli P, Rota MA, Alba S, Cuda G (2021). Proteomic profile of EPS-Urine through FASP digestion and data-independent analysis. J Visualized Exp.

[25] Huang T, Bruderer R, Muntel J, Xuan Y, Vitek O, Reiter L (2020). Combining precursor and fragment information for improved detection of differential abundance in data independent acquisition. Mol Cell Proteomics..

[26] Pham TV, Henneman AA, Jimenez CR (2020). iq: an R package to estimate relative protein abundances from ion quantification in DIA-MS-based proteomics. Bioinformatics..

[27] Ritchie ME, Phipson B, Wu D, Hu Y, Law CW, Shi W (2015). limma powers differential expression analyses for RNA-sequencing and microarray studies. Nucleic Acids Res.

[28] GSEABase: Gene set enrichment data structures and methods version 1.52.1 from Bioconductor [Internet]. [cited 2022 Aug 1]. Available from: https://rdrr.io/bioc/GSEABase/

[29] Yu G, Wang LG, Han Y, He QY (2012). clusterProfiler: an R package for comparing biological themes among gene clusters. OMICS..

[30] Yuan M, Breitkopf SB, Yang X, Asara JM (2012). A positive/negative ion–switching, targeted mass spectrometry–based metabolomics platform for bodily fluids, cells, and fresh and fixed tissue. Nat Protoc..

[31] Bajad SU, Lu W, Kimball EH, Yuan J, Peterson C, Rabinowitz JD (2006). Separation and quantitation of water soluble cellular metabolites by hydrophilic interaction chromatography-tandem mass spectrometry. J Chromatogr A.

[32] Mccloskey D, Ubhi BK. Quantitative and Qualitative Metabolomics for the Investigation of Intracellular Metabolism Targeted Analysis on the QTRAP ® 5500 System and Reverse-Phase Ion-Pairing Chromatography Key Features of the QTRAP ® 5500 System for Qualitative and Quantitative Metabolomics.

[33] Pang Z, Chong J, Zhou G, de Lima Morais DA, Chang L, Barrette M (2021). MetaboAnalyst 5.0: narrowing the gap between raw spectra and functional insights. Nucleic Acids Res..

[34] Zolea F, Battaglia AM, Chiarella E, Malanga D, de Marco C, Bond HM (2017). Ferritin heavy subunit silencing blocks the erythroid commitment of K562 cells via miR-150 up-regulation and GATA-1 repression. Int J Mol Sci.

[35] Zolea F, Biamonte F, Battaglia AM, Faniello MC, Cuda G, Costanzo F (2016). Caffeine positively modulates ferritin heavy chain expression in H460 cells: effects on cell proliferation. PLoS One..

[36] Battaglia AM, Sacco A, Perrotta ID, Faniello MC, Scalise M, Torella D (2022). Iron administration overcomes resistance to erastin-mediated ferroptosis in ovarian cancer cells. Front Oncol..

[37] Biamonte F, Buffone C, Santamaria G, Battaglia AM, Mignogna C, Fortunato L (2021). Gene expression analysis of autofluorescence margins in leukoplakia and oral carcinoma: a pilot study. Oral Dis..

[38] Biamonte F, Zolea F, Santamaria G, Battaglia AM, Cuda G, Costanzo F (2017). Human haematological and epithelial tumor-derived cell lines express distinct patterns of onco-microRNAs. Cell Mol Biol..

[39] Chirillo R, Aversa I, di Vito A, Salatino A, Battaglia AM, Sacco A (2020). FtH-Mediated ros dysregulation promotes CXCL12/CXCR4 axis activation and EMT-Like trans-differentiation in erythroleukemia K562 cells. Front Oncol.

[40] di Sanzo M, Aversa I, Santamaria G, Gagliardi M, Panebianco M, Biamonte F (2016). FTH1P3, a novel H-Ferritin pseudogene transcriptionally active, is ubiquitously expressed and regulated during cell Differ. PLoS One..

[41] Mendes Oliveira D, Grillone K, Mignogna C, de Falco V, Laudanna C, Biamonte F (2018). Next-generation sequencing analysis of receptor-type tyrosine kinase genes in surgically resected colon cancer: identification of gain-of-function mutations in the RET proto-oncogene. J Exp Clin Cancer Res..

[42] di Sanzo M, Chirillo R, Aversa I, Biamonte F, Santamaria G, Giovannone ED (2018). shRNA targeting of ferritin heavy chain activates H19/miR-675 axis in K562 cells. Gene.

[43] Bruschini S, di Martino S, Pisanu ME, Fattore L, de Vitis C, Laquintana V (2020). CytoMatrix for a reliable and simple characterization of lung cancer stem cells from malignant pleural effusions. J Cell Physiol..

[44] Yang KC, Gorski SM (2022). Protocol for analysis of RNA-sequencing and proteome profiling data for subgroup identification and comparison. STAR Protoc.

[45] Melissaridou S, Wiechec E, Magan M, Jain MV, Chung MK, Farnebo L (2019). The effect of 2D and 3D cell cultures on treatment response, EMT profile and stem cell features in head and neck cancer. Cancer Cell Int.

[46] Nowacka M, Sterzynska K, Andrzejewska M, Nowicki M, Januchowski R (2021). Drug resistance evaluation in novel 3D in vitro model. Biomed Pharmacother.

[47] Riffle S, Hegde RS (2017). Modeling tumor cell adaptations to hypoxia in multicellular tumor spheroids. J Exp Clin Cancer Res..

[48] Strainiene E, Binkis M, Urnikyte S, Stankevicius V, Sasnauskiene A, Kundrotas G (2018). Microenvironment dependent gene expression signatures in reprogrammed human colon normal and cancer cell lines. BMC Cancer..

[49] Park MC, Jeong H, Son SH, Kim YH, Han D, Goughnour PC (2016). Novel morphologic and genetic analysis of cancer cells in a 3D microenvironment identifies STAT3 as a regulator of tumor permeability barrier function. Cancer Res..

[50] Gendre DAJ, Ameti E, Karenovics W, Perriraz-Mayer N, Triponez F, Serre-Beinier V (2021). Optimization of tumor spheroid model in mesothelioma and lung cancers and anti-cancer drug testing in H2052/484 spheroids. Oncotarget.

[51] Panasyuk G, Espeillac C, Chauvin C, Pradelli LA, Horie Y, Suzuki A (2012). PPARγ contributes to PKM2 and HK2 expression in fatty liver. Nat Commun.

[52] Pirovich DB, Da‘dara AA, Skelly PJ (2021). Multifunctional fructose 1,6-bisphosphate aldolase as a therapeutic target. Front Mol Biosci..

[53] Haque A, Ray SK, Cox A, Banik NL (2016). Neuron specific enolase: a promising therapeutic target in acute spinal cord injury. Metab Brain Dis.

[54] Feng J, Li J, Wu L, Yu Q, Ji J, Wu J (2020). Emerging roles and the regulation of aerobic glycolysis in hepatocellular carcinoma. J Exp Clin Cancer Res..

[55] Cao L, Wu J, Qu X, Sheng J, Cui M, Liu S (2020). Glycometabolic rearrangements–aerobic glycolysis in pancreatic cancer: causes, characteristics and clinical applications. J Exp Clin Cancer Res..

[56] Sun Z, Tan Z, Peng C, Yi W (2021). HK2 is associated with the Warburg effect and proliferation in liver cancer: Targets for effective therapy with glycyrrhizin. Mol Med Rep..

[57] Tanner LB, Goglia AG, Wei MH, Sehgal T, Parsons LR, Park JO (2018). Four key steps control glycolytic flux in mammalian cells. Cell Syst.

[58] Bae E, Kim HE, Koh E, Kim KS (2014). Phosphoglucomutase1 is necessary for sustained cell growth under repetitive glucose depletion. FEBS Lett.

[59] Ivan M, Kaelin WG (2017). The EGLN-HIF O2 Sensing System: Multiple Inputs and Feedbacks. Mol Cell.

[60] Bellot G, Garcia-Medina R, Gounon P, Chiche J, Roux D, Pouysségur J (2009). Hypoxia-induced autophagy is mediated through hypoxia-inducible factor induction of BNIP3 and BNIP3L via their BH3 domains. Mol Cell Biol..

[61] Lupu IE, Redpath AN, Smart N (2021). SRSF3 is a key regulator of epicardial formation. bioRxiv..

[62] Zhang J, Ney PA (2009). Role of BNIP3 and NIX in cell death, autophagy, and mitophagy. Cell Death Differ..

[63] Kachhap SK, Faith D, Qian DZ, Shabbeer S, Galloway NL, Pili R (2007). The N-Myc down regulated Gene1 (NDRG1) Is a Rab4a effector involved in vesicular recycling of E-cadherin. PLoS One..

[64] Liu N, Wang L, Li X, Yang Q, Liu X, Zhang J (2008). N-Myc downstream-regulated gene 2 is involved in p53-mediated apoptosis. Nucleic Acids Res.

[65] VandeKopple MJ, Wu J, Auer EN, Giaccia AJ, Denko NC, Papandreou I (2019). HILPDA regulates lipid metabolism, lipid droplet abundance, and response to microenvironmental stress in solid tumors. Mol Cancer Res..

[66] Zhu Y, McAvoy S, Kuhn R, Smith DI (2006). RORA, a large common fragile site gene, is involved in cellular stress response. Oncogene..

[67] Zhan X, Zhong X, Choi JH, Su L, Wang J, Nair-Gill E (2020). Adenosine monophosphate deaminase 3 null mutation causes reduction of naive T cells in mouse peripheral blood. Blood Adv.

[68] Nemkov T, Sun K, Reisz JA, Song A, Yoshida T, Dunham A (2018). Hypoxia modulates the purine salvage pathway and decreases red blood cell and supernatant levels of hypoxanthine during refrigerated storage. Haematologica.

[69] Hardie DG (2011). AMP-activated protein kinase—an energy sensor that regulates all aspects of cell function. Genes Dev.

[70] Jeon SM, Chandel NS, Hay N (2012). AMPK regulates NADPH homeostasis to promote tumour cell survival during energy stress. Nature.

[71] Song L, Li XX, Liu XY, Wang Z, Yu Y, Shi M (2020). EFEMP2 suppresses the invasion of lung cancer cells by inhibiting Epithelial-Mesenchymal Transition (EMT) and down-regulating MMPs. Onco Targets Ther.

[72] Mao W, Wang K, Wu Z, Xu B, Chen M (2021). Current status of research on exosomes in general, and for the diagnosis and treatment of kidney cancer in particular. J Exp Clin Cancer Res..

[73] Wu W, Zheng X, Wang J, Yang T, Dai W, Song S (2018). O-GlcNAcylation on Rab3A attenuates its effects on mitochondrial oxidative phosphorylation and metastasis in hepatocellular carcinoma. Cell Death Dis.

[74] de Silva D, Tu YT, Amunts A, Fontanesi F, Barrientos A (2015). Mitochondrial ribosome assembly in health and disease. Cell Cycle.

[75] di Nottia M, Marchese M, Verrigni D, Mutti CD, Torraco A, Oliva R (2020). A homozygous MRPL24 mutation causes a complex movement disorder and affects the mitoribosome assembly. Neurobiol Dis.

[76] Ludwig-Galezowska AH, Flanagan L, Rehm M (2011). Apoptosis repressor with caspase recruitment domain, a multifunctional modulator of cell death. J Cell Mol Med.

[77] Bravo-San Pedro JM, Sica V, Martins I, Pol J, Loos F, Maiuri MC (2019). Acyl-CoA-binding protein is a lipogenic factor that triggers food intake and obesity. Cell Metab..

[78] Szigeti A, Bellyei S, Gasz B, Boronkai A, Hocsak E, Minik O (2006). Induction of necrotic cell death and mitochondrial permeabilization by heme binding protein 2/SOUL. FEBS Lett..

[79] Yin L, Xiang Y, Zhu DY, Yan N, Huang RH, Zhang Y (2005). Crystal structure of human SH3BGRL protein: The first structure of the human SH3BGR family representing a novel class of thioredoxin fold proteins. Proteins..

[80] Gry M, Rimini R, Strömberg S, Asplund A, Pontén F, Uhlén M (2009). Correlations between RNA and protein expression profiles in 23 human cell lines. BMC Genomics..

[81] Menendez JA, Alarco NT (2014). Metabostemness: A new cancer hallmark. Front Oncol..

[82] Wu M, Neilson A, Swift AL, Moran R, Tamagnine J, Parslow D (2007). Multiparameter metabolic analysis reveals a close link between attenuated mitochondrial bioenergetic function and enhanced glycolysis dependency in human tumor cells. Am J Physiol Cell Physiol..

[83] Louie MC, Ton J, Brady ML, Le DT, Mar JN, Lerner CA (2020). Total cellular ATP production changes with primary substrate in MCF7 breast cancer cells. Front Oncol.

[84] Edmondson R, Broglie JJ, Adcock AF, Yang L (2014). Three-dimensional cell culture systems and their applications in drug discovery and cell-based biosensors. Assay Drug Dev Technol.

[85] Eslami-S Z, Cortés-Hernández LE, Thomas F, Pantel K, Alix-Panabières C (2022). Functional analysis of circulating tumour cells: the KEY to understand the biology of the metastatic cascade. Brit J Cancer..

[86] van Zijl F, Krupitza G, Mikulits W (2011). Initial steps of metastasis: cell invasion and endothelial transmigration. Mutat Res.

[87] Paoli P, Giannoni E, Chiarugi P (2013). Anoikis molecular pathways and its role in cancer progression. Biochim et Biophys Acta (BBA).

[88] Walsh N, Clynes M, Crown J, O’Donovan N (2009). Alterations in integrin expression modulates invasion of pancreatic cancer cells. J Exp Clin Cancer Res..

[89] Cheng Y, Hou T, Ping J, Chen T, Yin B (2018). LMO3 promotes hepatocellular carcinoma invasion, metastasis and anoikis inhibition by directly interacting with LATS1 and suppressing Hippo signaling 06 biological sciences 0601 biochemistry and cell biology. J Exp Clin Cancer Res..

[90] Ghoneum A, Abdulfattah AY, Warren BO, Shu J, Said N (2020). Redox homeostasis and metabolism in cancer: a complex mechanism and potential targeted therapeutics. Int J Mol Sci..

[91] Yoshida GJ (2015). Metabolic reprogramming: the emerging concept and associated therapeutic strategies. J Exp Clin Cancer Res..

[92] Rios Garcia M, Steinbauer B, Srivastava K, Singhal M, Mattijssen F, Maida A (2017). Acetyl-CoA Carboxylase 1-dependent protein acetylation controls breast cancer metastasis and recurrence. Cell Metab..

[93] Chuang CH, Dorsch M, Dujardin P, Silas S, Ueffing K, Holken JM (2021). Altered mitochondria functionality defines a metastatic cell state in lung cancer and creates an exploitable vulnerability. Cancer Res..

[94] Jin L, Chun J, Pan C, Alesi GN, Li D, Magliocca KR (2017). Phosphorylation-mediated activation of LDHA promotes cancer cell invasion and tumour metastasis. Oncogene..

[95] Zera K, Zastre J (2018). Stabilization of the hypoxia-inducible transcription Factor-1 alpha (HIF-1α) in thiamine deficiency is mediated by pyruvate accumulation. Toxicol Appl Pharmacol..

[96] Xiang L, Mou J, Shao B, Wei Y, Liang H, Takano N (2019). Glutaminase 1 expression in colorectal cancer cells is induced by hypoxia and required for tumor growth, invasion, and metastatic colonization. Cell Death Dis..

[97] Liu G, Zhu J, Yu M, Cai C, Zhou Y, Yu M (2015). Glutamate dehydrogenase is a novel prognostic marker and predicts metastases in colorectal cancer patients. J Transl Med..

[98] Ran H, Zhu Y, Deng R, Zhang Q, Liu X, Feng M (2018). Stearoyl-CoA desaturase-1 promotes colorectal cancer metastasis in response to glucose by suppressing PTEN. J Exp Clin Cancer Res..

[99] Nie J, Zhang J, Wang L, Lu L, Yuan Q, An F (2017). Adipocytes promote cholangiocarcinoma metastasis through fatty acid binding protein 4. J Exp Clin Cancer Res..

[100] Yi M, Li J, Chen S, Cai J, Ban Y, Peng Q (2018). Correction to: Emerging role of lipid metabolism alterations in cancer stem cells. J Exp Clin Cancer Res..

[101] Pisanu ME, Maugeri-Saccà M, Fattore L, Bruschini S, de Vitis C, Tabbì E (2018). Inhibition of Stearoyl-CoA desaturase 1 reverts BRAF and MEK inhibition-induced selection of cancer stem cells in BRAF-mutated melanoma. J Exp Clin Cancer Res..

[102] Sorrentino G, Ruggeri N, Specchia V, Cordenonsi M, Mano M, Dupont S (2014). Metabolic control of YAP and TAZ by the mevalonate pathway. Nature Cell Biol..

[103] Kubik J, Humeniuk E, Adamczuk G, Madej-Czerwonka B, Korga-Plewko A (2022). Targeting energy metabolism in cancer treatment. Int J Mol Sci.

[104] Yoshida GJ (2015). Metabolic reprogramming: The emerging concept and associated therapeutic strategies. J Exp Clin Cancer Res..

[105] Lin J, Xia L, Liang J, Han Y, Wang H, Oyang L (2019). The roles of glucose metabolic reprogramming in chemo- and radio-resistance. J Exp Clin Cancer Res..

[106] Liberti MV, Locasale JW (2016). The Warburg effect: how does it benefit cancer cells?. Trends Biochem Sci..

[107] Mishra D, Banerjee D (2019). Lactate dehydrogenases as metabolic links between tumor and stroma in the tumor microenvironment. Cancers (Basel).

[108] Liu S, Zhao H, Hu Y, Yan C, Mi Y, Li X (2022). Lactate promotes metastasis of normoxic colorectal cancer stem cells through PGC-1α-mediated oxidative phosphorylation. Cell Death Dis.

[109] Tang L, Wei F, Wu Y, He Y, Shi L, Xiong F (2018). Role of metabolism in cancer cell radioresistance and radiosensitization methods. J Exp Clin Cancer Res..

[110] Wang W, Ru J, Hu C, Chen C, Quan ZH, Wang AL (2021). en C, Quan ZH, Wang AL. et al. Pharmacologically inhibiting phosphoglycerat. Acta Pharmacol Sin..

[111] Ishii A, Kimura T, Sadahiro H, Kawano H, Takubo K, Suzuki M (2016). Histological characterization of the tumorigenic “peri-necrotic niche” harboring quiescent stem-like tumor cells in glioblastoma. PLoS One..

[112] Walenta S, Dotsch J, Bourrat-Flock B, Mueller-Klieser W (1990). Size-dependent oxygenation and energy status in multicellular tumor spheroids. Adv Exp Med Biol..

[113] de Gaetano A, Gibellini L, Zanini G, Nasi M, Cossarizza A, Pinti M (2021). Mitophagy and oxidative stress: the role of aging. Antioxidants.

[114] Castelli S, Ciccarone F, Tavian D, Ciriolo MR (2021). ROS-dependent HIF1α activation under forced lipid catabolism entails glycolysis and mitophagy as mediators of higher proliferation rate in cervical cancer cells. J Exp Clin Cancer Res.

[115] Semenza GL (2013). HIF-1 mediates metabolic responses to intratumoral hypoxia and oncogenic mutations. J Clin Invest.

[116] Nakagawa T, Lanaspa MA, Millan IS, Fini M, Rivard CJ, Sanchez-Lozada LG (2020). Fructose contributes to the Warburg effect for cancer growth. Cancer Metab.

[117] Tappy L, Rosset R (2019). Health outcomes of a high fructose intake: the importance of physical activity. J Physiol.

[118] Nakagawa T, Kang DH (2021). Fructose in the kidney: from physiology to pathology. Kidney Res Clin Pract.

[119] Kushiyama A, Nakatsu Y, Matsunaga Y, Yamamotoya T, Mori K, Ueda K (2016). Role of uric acid metabolism-related inflammation in the pathogenesis of metabolic syndrome components such as atherosclerosis and nonalcoholic steatohepatitis. Mediators Inflamm.

[120] Hardie DG, Lin SC. AMP-activated protein kinase - not just an energy sensor. F1000Res. 2017;6:1724. 10.12688/f1000research.11960.1. 10.12688/f1000research.11960.1 PMC5615778 29034085

[121] Pan C, Li B, Simon MC (2021). Moonlighting functions of metabolic enzymes and metabolites in cancer. Mol Cell.

[122] Lew CR, Tolan DR (2012). Targeting of several glycolytic enzymes using RNA interference reveals aldolase affects cancer cell proliferation through a non-glycolytic mechanism. J Biol Chem.

[123] Wang V, Davis DA, Haque M, Huang LE, Yarchoan R (2005). Differential gene up-regulation by hypoxia-inducible factor-1α and hypoxia-inducible factor-2α in HEK293T Cells. Cancer Res..

[124] Maruyama R, Nagaoka Y, Ishikawa A, Akabane S, Fujiki Y, Taniyama D (2022). Overexpression of aldolase, fructose-bisphosphate C and its association with spheroid formation in colorectal cancer. Pathol Int.

[125] Zha Z, Li D, Zhang P, Wang P, Fang X, Liu X (2021). Neuron specific enolase promotes tumor metastasis by activating the Wnt/β-catenin pathway in small cell lung cancer. Transl Oncol.

[126] Dumitrescu C, Ameye L, Latifyan S, Elali Z, Lossignol D, Dumitrescu C (2017). Neuron specific enolase, a biomarker of breast cancer cerebral metastasis. Open Access Library J Sci.

[127] Muoio B, Pascale M, Roggero E (2018). The role of serum neuron-specific enolase in patients with prostate cancer: a systematic review of the recent literature. Int J Biol Markers.

[128] Yukimoto R, Nishida N, Hata T, Fujino S, Ogino T, Miyoshi N (2021). Specific activation of glycolytic enzyme enolase 2 in BRAF V600E-mutated colorectal cancer. Cancer Sci.

[129] Yu Z, Li Q, An Y, Chen X, Liu Z, Li Z (2019). Role of apoptosis repressor with caspase recruitment domain (ARC) in cancer (Review). Oncol Lett..

