# Supplementary material for: ALDOC- and ENO2- driven glucose metabolism sustains 3D tumor spheroids growth regardless of nutrient environmental conditions: a multi-omics analysis
Source: J Exp Clin Cancer Res. 2023 Mar 22;42:69. doi: 10.1186/s13046-023-02641-0 (PMC10031988; doi:10.1186/s13046-023-02641-0)
Supplement: Supplementary file 6 — Additional file 6: Figure S1. Correlations between mRNA expression and protein abundance. Sample-wise mRNA-protein correlation computed as Spearman’s Rho (y-axis). Samples are ordered along the x-axis based on increasing correlation. [file 13046_2023_2641_MOESM6_ESM.docx]

**Additional File 6**


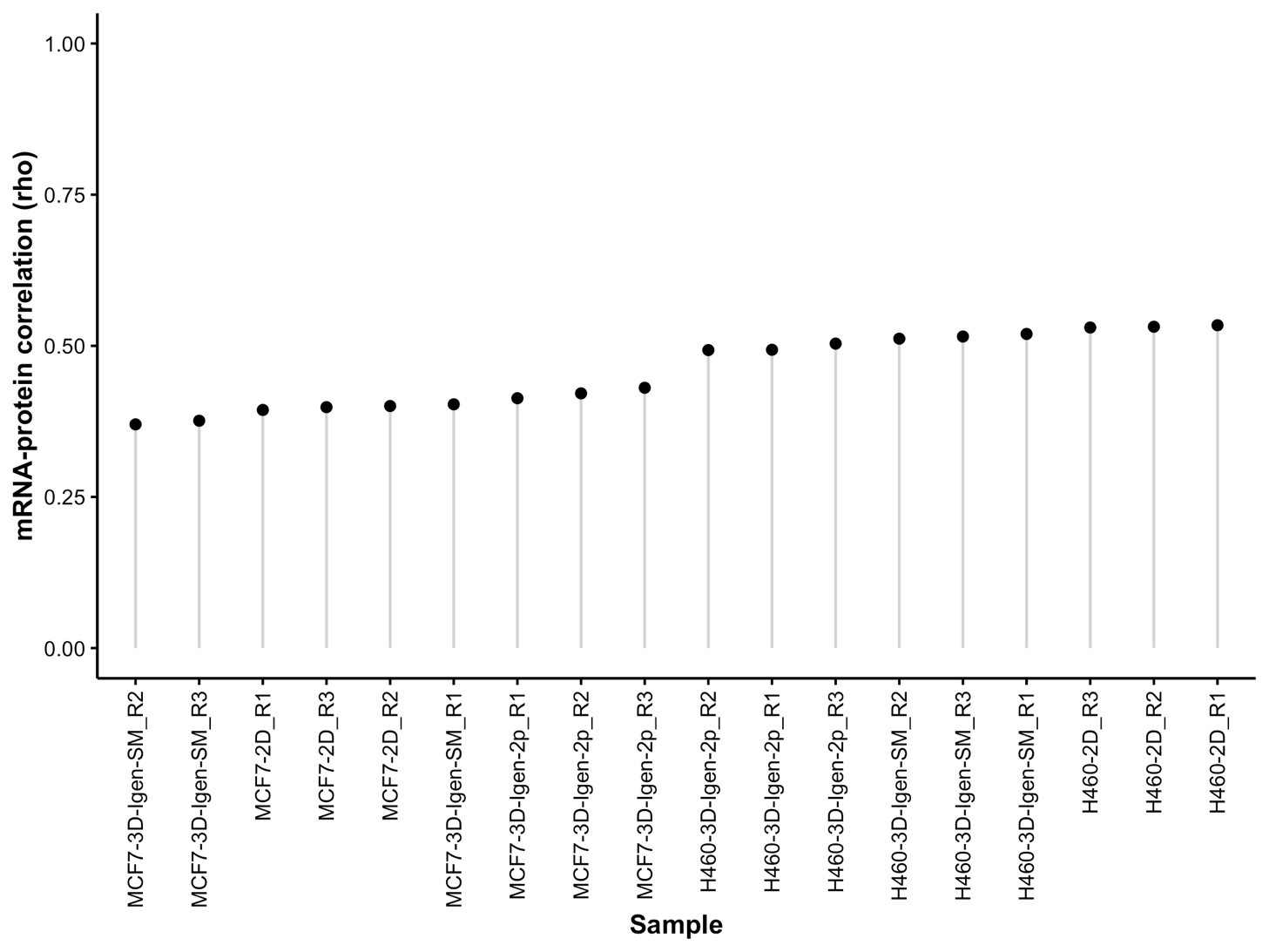
**Figure S1**: Correlations between mRNA expression and protein abundance.

Sample-wise mRNA-protein correlation computed as Spearman’s Rho (y-axis). Samples are ordered along the x-axis based on increasing correlation.
