# Supplementary material for: ALDOC- and ENO2- driven glucose metabolism sustains 3D tumor spheroids growth regardless of nutrient environmental conditions: a multi-omics analysis
Source: J Exp Clin Cancer Res. 2023 Mar 22;42:69. doi: 10.1186/s13046-023-02641-0 (PMC10031988; doi:10.1186/s13046-023-02641-0)
Supplement: Supplementary file 8 — Additional file 8: Figure S2. ALDOC and ENO2 levels increase in all H460 and MCF7 3D conditions compared to their 2D relative counterparts. A-B qRT-PCR and Western Blot analyses of ALDOC and ENO2 in H460 2D, H460 3D_SM, H460 3D_FBSlow, MCF7 2D, MCF7 3D_SM, and MCF7 3D_FBSlow. All the experiments were carried out in triplicate and results are presented as mean ± SD. p-value: *<0.05. [file 13046_2023_2641_MOESM8_ESM.docx]

**Additional File 8**

**Figure S2**: ALDOC and ENO2 levels increase in all H460 and MCF7 3D conditions compared to their 2D relative counterparts.


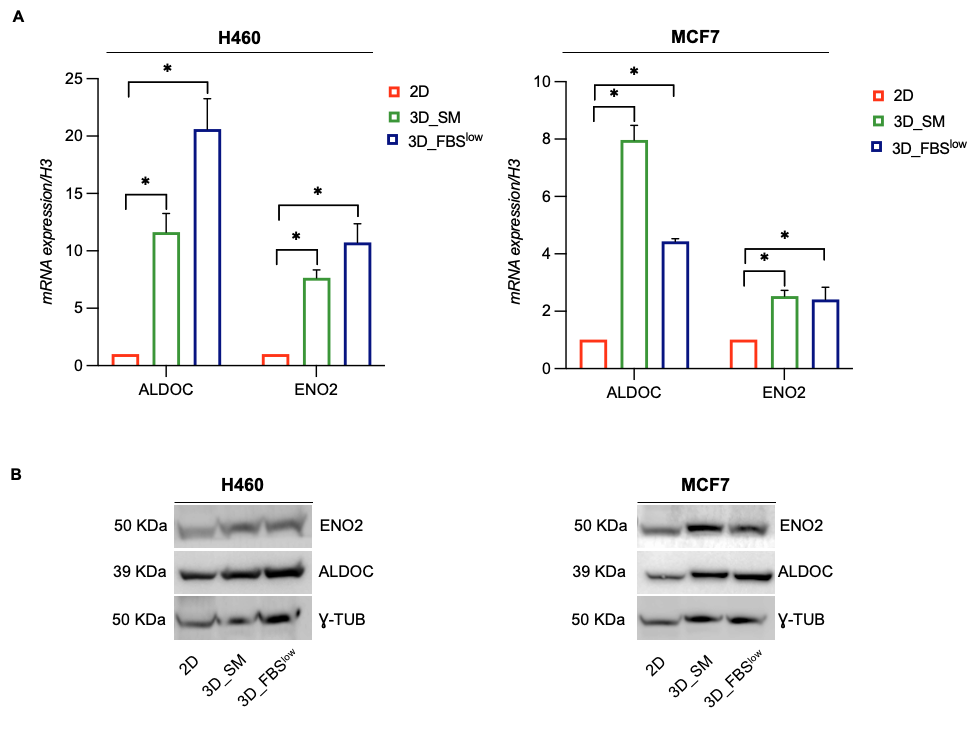


**Figure S2**: ALDOC and ENO2 levels increase in all H460 and MCF7 3D conditions compared to their 2D relative counterparts. **A-B** qRT-PCR and Western Blot analyses of ALDOC and ENO2 in H460 2D, H460 3D_SM, H460 3D_FBS^low^, MCF7 2D, MCF7 3D_SM, and MCF7 3D_FBS^low^. All the experiments were carried out in triplicate and results are presented as mean ± SD. *p-*value: *<0.05.
