# Supplementary material for: ALDOC- and ENO2- driven glucose metabolism sustains 3D tumor spheroids growth regardless of nutrient environmental conditions: a multi-omics analysis
Source: J Exp Clin Cancer Res. 2023 Mar 22;42:69. doi: 10.1186/s13046-023-02641-0 (PMC10031988; doi:10.1186/s13046-023-02641-0)

**Additional File 9**

**Figure S3**: Schematic illustration of upstream and downstream events of *ALDOC* and/or *ENO2* perturbation


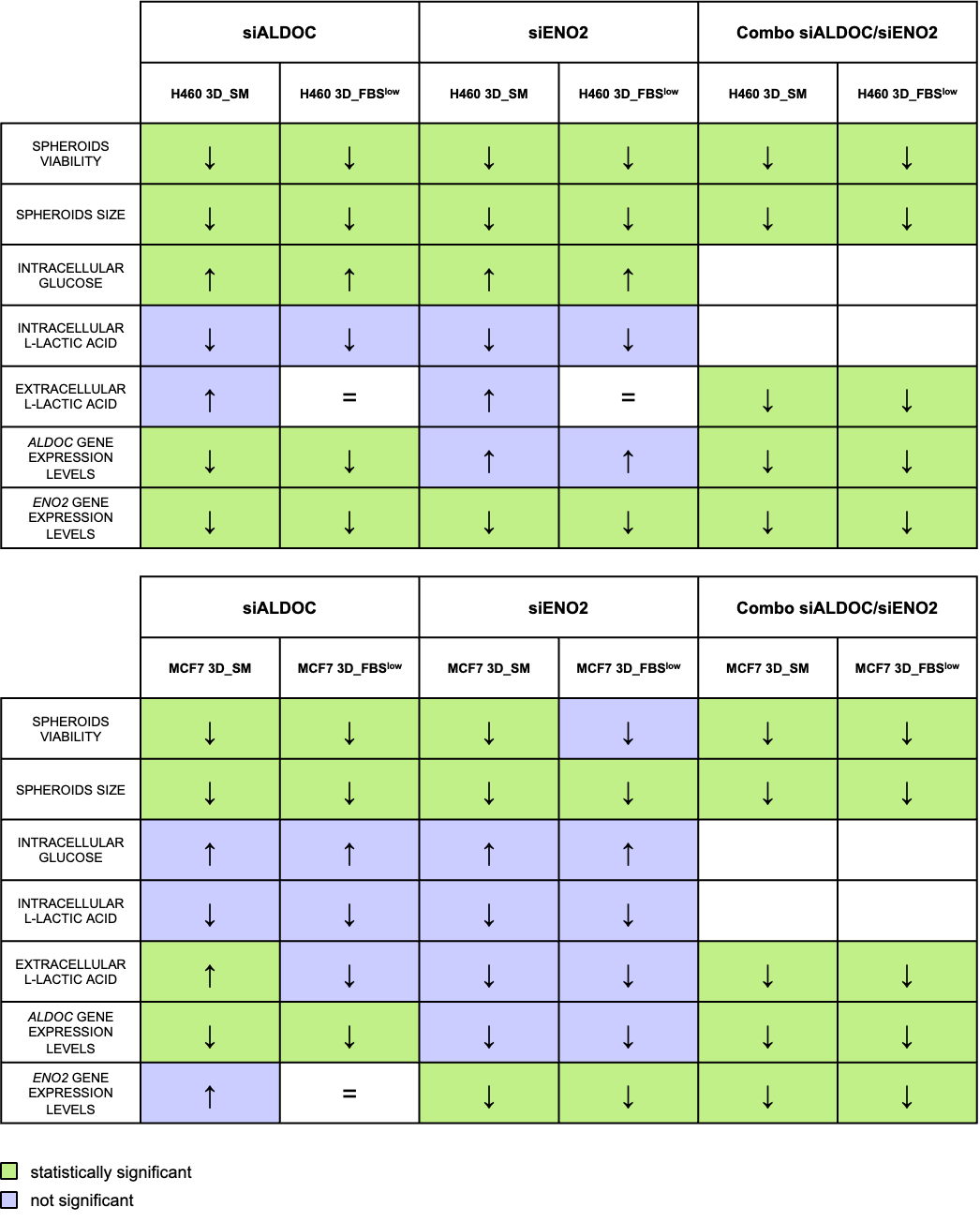

Supplement: Supplementary file 9 — Additional file 9: Figure S3. Schematic illustration of upstream and downstream events of ALDOC and/or ENO2 perturbation. [file 13046_2023_2641_MOESM9_ESM.docx]
