# Supplementary material for: ALDOC- and ENO2- driven glucose metabolism sustains 3D tumor spheroids growth regardless of nutrient environmental conditions: a multi-omics analysis
Source: J Exp Clin Cancer Res. 2023 Mar 22;42:69. doi: 10.1186/s13046-023-02641-0 (PMC10031988; doi:10.1186/s13046-023-02641-0)
Supplement: Supplementary file 10 — Additional file 10: Fig. S4. ALDOC and ENO2 knock down with additional siRNAs confirms the reduction of sphere-forming ability of H460 and MCF7 cells. A qRT-PCR analyses of ALDOC and ENO2 in H460 3D_SM, H460 3D_FBSlow, MCF7 3D_SM, and MCF7 3D_FBSlow upon ALDOC and ENO2 silencing with additional siRNAs. B Cell viability of H460 3D_SM, H460 3D_FBSlow, T47D 3D_SM, and T47D 3D_FBSlow upon ALDOC and ENO2 silencing with additional siRNAs assessed by Cell titer-Glo 3D assay and expressed as relative light unit (R.L.U.). D Representative images and relative histograms of tumor spheroids morphology and diameter of H460 3D_SM, H460 3D_FBSlow, MCF7 3D_SM, and MCF7 3D_FBSlow upon ALDOC and ENO2 silencing with additional siRNAs. All the experiments were carried out in triplicate and results are presented as mean ± SD. p-value: *<0.05, **<0.01, ***<0.001. ns: not significant. [file 13046_2023_2641_MOESM10_ESM.docx]

**Additional File 10**


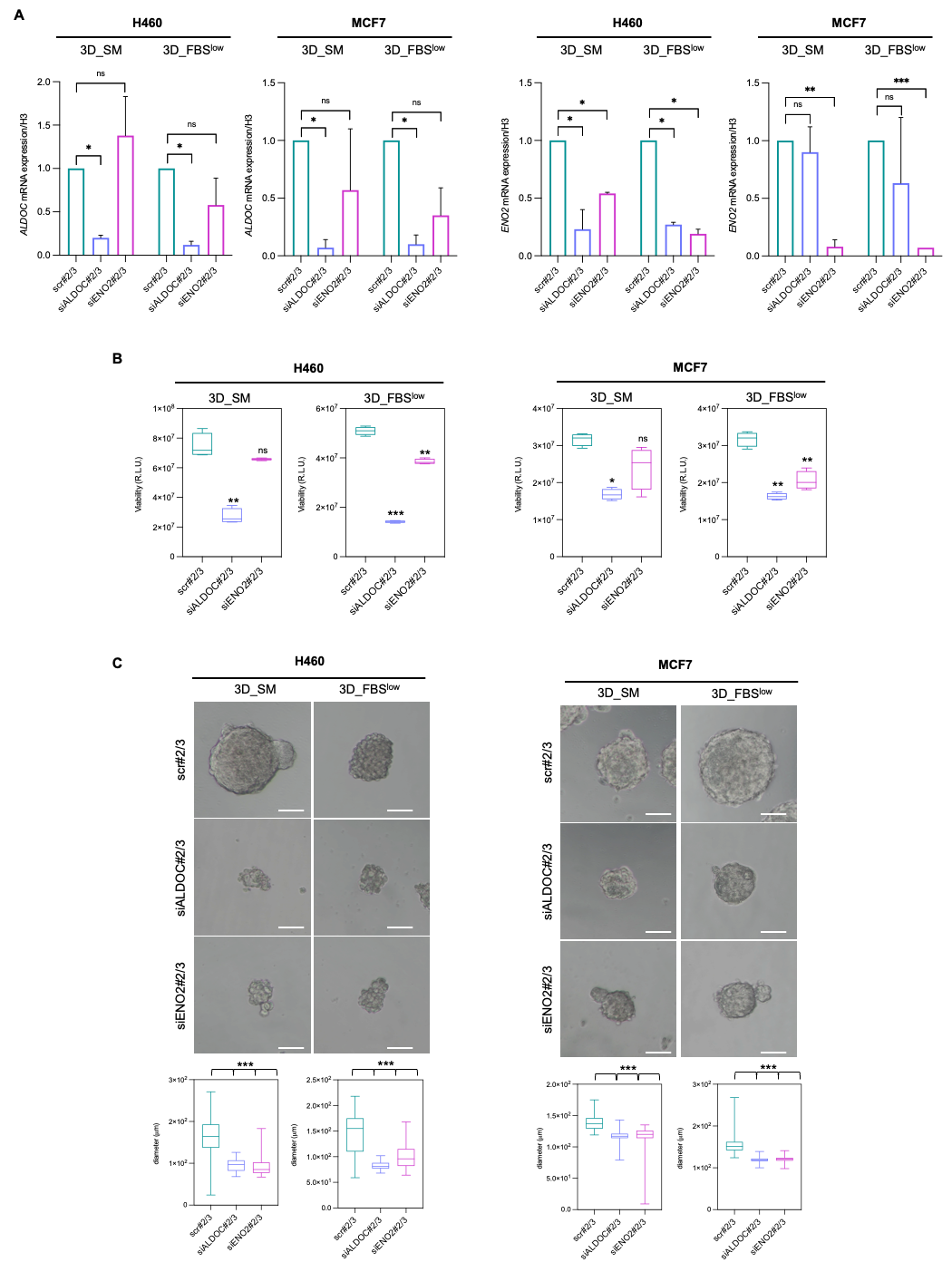
**Figure S4**: *ALDOC* and *ENO2* knock down with additional siRNAs confirms the reduction of sphere-forming ability of H460 and MCF7 cells.

**Fig. S4** *ALDOC* and *ENO2* knock down with additional siRNAs confirms the reduction of sphere-forming ability of H460 and MCF7 cells. **A** qRT-PCR analyses of *ALDOC* and *ENO2* in H460 3D_SM, H460 3D_FBS^low^, MCF7 3D_SM, and MCF7 3D_FBS^low^ upon *ALDOC* and *ENO2* silencing with additional siRNAs. **B** Cell viability of H460 3D_SM, H460 3D_FBS^low^, T47D 3D_SM, and T47D 3D_FBS^low^ upon *ALDOC* and *ENO2* silencing with additional siRNAs assessed by Cell titer-Glo 3D assay and expressed as relative light unit (R.L.U.). **D** Representative images and relative histograms of tumor spheroids morphology and diameter of H460 3D_SM, H460 3D_FBS^low^, MCF7 3D_SM, and MCF7 3D_FBS^low^ upon *ALDOC* and *ENO2* silencing with additional siRNAs. All the experiments were carried out in triplicate and results are presented as mean ± SD. *p-*value: *<0.05, **<0.01, ***<0.001. ns: not significant.
