# Supplementary material for: ALDOC- and ENO2- driven glucose metabolism sustains 3D tumor spheroids growth regardless of nutrient environmental conditions: a multi-omics analysis
Source: J Exp Clin Cancer Res. 2023 Mar 22;42:69. doi: 10.1186/s13046-023-02641-0 (PMC10031988; doi:10.1186/s13046-023-02641-0)
Supplement: Supplementary file 11 — Additional file 11: Fig. S5. ALDOC and ENO2 knock down with additional siRNAs also impairs glucose and L-lactic acid amounts in H460 and MCF7 3D tumor spheroids. A-B Intracellular glucose and L-lactic acid amounts measured by luminometric assays and reported as relative light units (R.L.U.) in H460 3D_ SM, H460 3D_FBSlow, MCF7 3D_SM and MCF7 3D_FBSlow upon ALDOC and ENO2 silencing with additional siRNAs. C Quantification of L-lactic acid within the culture media (extracellular) performed by emogas analysis and expressed as mmol/1 in H460 3D_SM, H460 3D_ FBSlow, MCF7 3D_SM and MCF7 3D_ FBSlow upon ALDOC and ENO2 silencing with additional siRNAs. All the experiments were carried out in triplicate and results are presented as mean = SD. ns: not significant. [file 13046_2023_2641_MOESM11_ESM.docx]

**Additional File 11**

**Figure S5**: *ALDOC* and *ENO2* knock down with additional siRNAs also impairs glucose and L-lactic acid amounts in H460 and MCF7 3D tumor spheroids.


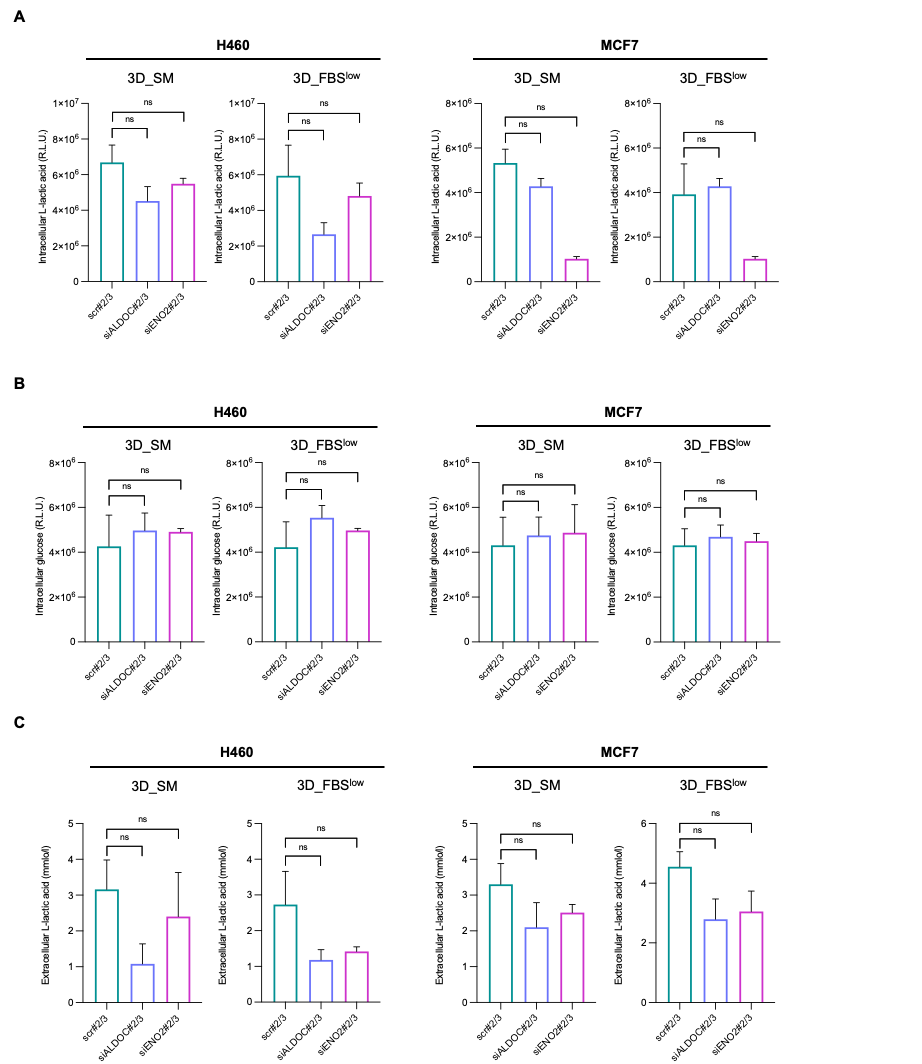


**Fig. S5** *ALDOC* and *ENO2* knock down with additional siRNAs also impairs glucose and L-lactic acid amounts in H460 and MCF7 3D tumor spheroids. **A-B** Intracellular glucose and L-lactic acid amounts measured by luminometric assays and reported as relative light units (R.L.U.) in H460 3D_ SM, H460 3D_FBS^low^, MCF7 3D_SM and MCF7 3D_FBS^low^ upon *ALDOC* and *ENO2* silencing with additional siRNAs. **C** Quantification of L-lactic acid within the culture media (extracellular) performed by emogas analysis and expressed as mmol/1 in H460 3D_SM, H460 3D_ FBS^low^, MCF7 3D_SM and MCF7 3D_ FBS^low^ upon *ALDOC* and *ENO2* silencing with additional siRNAs. All the experiments were carried out in triplicate and results are presented as mean = SD. ns: not significant.
